# An Agent-Based Modeling Dynamic Hybrid Model for Project Management in Research and Development

**DOI:** 10.1021/acs.iecr.5c04351

**Published:** 2026-02-26

**Authors:** Robson Wilson Silva Pessoa, Marie Hahn Naess, Julia Carolina Bijos, Carine Menezes Rebello, Danilo Colombo, Leizer Schnitman, Idelfonso B. R. Nogueira

**Affiliations:** † Department of Chemical Engineering, 8018 Norwegian University of Science and Technology, Trondheim 793101, Norway; ‡ CENPES, Petrobras R&D Center, Av. Horácio Macedo 950, Cid. Universitária, Ilha do Fundão, Salvador 21941-915, Brazil; § Postgraduate Program in Mechatronics, Federal University of Bahia, Polytechnic School, R. Prof. Aristídes Novis, 2, Federação, Salvador 40210-630, Brazil

## Abstract

This paper presents a hybrid approach to predict the evolution of technological maturity of R&D projects, using the context of the oil and gas (O&G) sector as an example. Integrating System Dynamics (SD) and Agent-based Modeling (ABM) enables the proposed multilevel framework to capture uncertainties inherent to R&D projects, including work effort, team size, and project duration, all of which influence technological progress. Although AB–SD hybrid models are well established in other fields, their application in R&D contexts remains limited. The AB–SD model combines system-level feedback structures governing work phases, rework cycles, and project duration with the explicit representation of decentralized agents (e.g., team members, tasks, and controllers) whose interactions drive emergent project dynamics. A base-case scenario was developed to analyze the structural dynamics of early-stage innovation projects, simulating 15 parallel tasks over 156 weeks. In a comparative scenario with sequential task execution, the model showed an 88% reduction in rework duration relative to the base case. The second scenario evaluated mixed parallel–sequential task structures under varying team sizes. In parallel configuration, simulation results indicated that increasing team size reduced overall project duration and improved task completion rates, with optimal performance achieved for teams of four to five members. These outcomes are consistent with empirical observations in R&D project management, where moderate team expansion enhances coordination efficiency without incurring communication overhead. However, as widely recognized in empirical studies, a substantial increase in team size does not necessarily translate into higher completion rates, as excessive team growth often introduces communication complexity and management delays. Overall, the model outputs and the proposed modeling framework are well aligned with expert understanding in the field, confirming their validity as a quantitative tool for analyzing resource allocation, task scheduling efficiency, and technology maturity progression.

## Graphical abstract

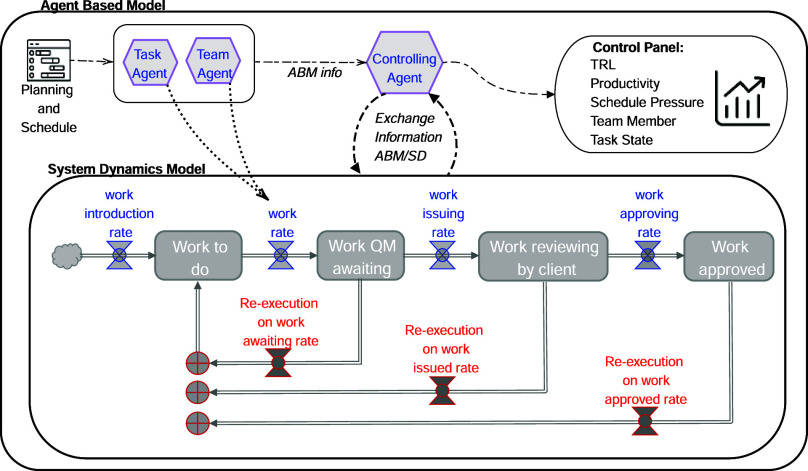

## 1 Introduction

Over the past decade, energy-transition policies have increasingly driven industry to enhance both the economic and environmental performance of its processes.^1^ This transformation is shaped by technological advancements, as well as the evolving organization of work and the coordination of innovation activities. Within this systemic view, Hekkert and Negro^2^ identify seven key functions of technological innovation systems–entrepreneurial activities, knowledge development, knowledge diffusion through networks, direction of research, market formation, mobilization of human and financial resources, and the creation of legitimacy to overcome resistance to change—which together underpin the management of technological and institutional transitions.

Within R&D environments, these innovation functions materialize through the interaction of multiple actors involved in planning, executing, and coordinating research activities. Building on the innovation systems framework, Klessova et al.^3^ analyzed European research and innovation programmes spanning different levels of technology maturity and showed how project management practices are closely linked to mechanisms of knowledge integration and resource mobilization. Their results highlight the inherently multiactor nature of R&D coordination, where diverse stakeholders interact to align knowledge flows, funding, and project objectives throughout the innovation lifecycle.

This systemic complexity is particularly evident in large energy companies, which adopt diverse implementation strategies and foster interactions across innovation functions through organizational arrangements that connect scientific, technological, and industrial institutions. The oil and gas sector offers a relevant example, as firms face the dual challenge of maintaining a reliable energy supply while accelerating the adoption of low-carbon technologies under increasingly stringent environmental regulations. These pressures intensify the need for structured R&D frameworks and cross-sectoral collaboration to manage uncertainty, coordination, and technological progression. In such settings, both qualitative and quantitative data can be leveraged to model R&D projects governed by emergent interactions among innovation functions, enabling a more systematic representation of technology development under energy transition constraints.

In line with the systemic and multiactor nature of R&D environments discussed above, structural modeling approaches capable of representing interdependencies and feedback mechanisms are required to describe technological progress comprehensively. Such approaches enable the explicit representation of cause–effect relationships among interconnected variables, providing a systemic understanding of technological innovation processes.^4^ Within this methodological landscape, System Dynamics (SD) enables the simultaneous modeling of technological, financial, policy, project management, and sustainability dimensions by explicitly representing feedback structures and nonlinear behaviors through causal loop and stock-and-flow formulations.^5^ It demonstrated how SD can be integrated with agent-based approaches to capture interactions between entities, while^6^ and^7^ illustrated its application to project performance assessment and policy evaluation in engineering contexts.

Concurrently, Agent-Based Modeling (ABM) complements SD by describing systems as populations of autonomous agents that operate under behavioral rules and interact within a shared environment. These local interactions give rise to emergent patterns that reflect the collective behavior of the system.^8–10^ This approach has been successfully used to simulate knowledge diffusion Schlaile et al.,^11^ to analyze policy strategies for innovation diffusion, and to examine the influence of subsidies in knowledge exchange.^12^ Within R&D contexts, agents can represent individuals or teams who execute research tasks, exchange information, and adapt their behavior in response to evolving project conditions such as funding availability, uncertainty, and technological barriers.

Despite these advances, there is still no established application of such hybrid modeling approaches in early-stage industrial R&D projects. Modeling in this setting involves specific challenges, as it must account for the high uncertainty, multilevel decision-making, and asynchronous task execution that characterize innovation processes, while capturing their interaction with technology maturity progression. Integrating the microlevel behavior of agents, such as teams and tasks, with the macro-level feedback structures that govern coordination, rework, and resource allocation remains a central challenge. Addressing this gap requires a modeling framework capable of coupling agent-level execution dynamics with system-level feedback mechanisms in a transparent and interpretable manner.

Within such a framework, a consistent and operational representation of technological maturity is required to link project execution dynamics with technology development outcomes. The concept of Technology Readiness Level (TRL) proposes a scale to quantify the progression of technological maturity from basic principles to fully operational systems. Over time, the framework has been adapted from the original NASA proposition to a wide range of industries, including the energy sector, where it has been applied to assess technology evolution, investment risk, and deployment readiness.^13,14^

In this context, approaches such as that developed by Kenley et al.^15^ illustrate how TRL can be reformulated into a quantitative and probabilistic representation of technology maturation, providing a foundation that can be further extended to other industrial sectors and innovation contexts. Kenley et al.^15^ introduced a statistical method to estimate the probability of TRL evolution using historical data from NASA and the U.S. Department of Energy, while Pujotomo et al.^16^ modeled the innovation ecosystem as an interacting network of projects positioned at different TRL levels. The latter demonstrated that analyzing these interconnections can reveal pathways to accelerate technology commercialization and to align research progress with market deployment objectives.

Building on these insights, system analysis has advanced our understanding of factors influencing technological progress and innovation performance. Nevertheless, the inherent complexity of such systems is not fully captured by conventional SD models, which operate primarily at the aggregate level. This limitation has motivated the adoption of complementary approaches that enable a multilevel representation of innovation ecosystems. Microlevel modeling, in particular, allows the explicit representation of agents and their interactions, capturing heterogeneity, nonlinearity, and spatial relationships across time.^17^ Although hybrid frameworks that combine SD with ABM have demonstrated significant potential in other domains, such as technology diffusion, health policy, and environmental systems,^17–19^ their application to the planning, monitoring, and management of industrial R&D processes remains scarce in the literature.

The integration of ABM with SD combines the strengths of both paradigms, linking continuous system-level feedback with discrete agent-level interactions. SD effectively models aggregated dynamics over time, yet it cannot explicitly represent task-level dependencies, sequential processes, or heterogeneous decision-making.^20^ ABM, conversely, captures these aspects through decentralized, rule-based interactions but lacks the formal mathematical representation of continuous feedback that SD provides.^9^ Merging these approaches enables the simultaneous simulation of microlevel execution dynamics and macro-level performance within a unified framework.

This study introduces a hybrid, dynamic, and predictive modeling framework that integrates SD and ABM to address the limitations of existing approaches in modeling industrial R&D projects. The proposed model links agent-level behaviors with system-level feedback structures, enabling the endogenous representation of project execution dynamics and technological maturity progression. A key feature of the framework is its ability to predict Technology Readiness Level (TRL) evolution as an emergent outcome of coordination, rework, and scheduling processes within R&D projects.

## 2 Methodology

The methodology developed in this study establishes a hybrid SD and ABM framework designed to represent the complex dynamics of R&D project execution and its relationship to technology maturity progression. The approach follows a multilayered structure that integrates macro-level feedback mechanisms with microlevel behavioral interactions. The first stage involves constructing the SD model, which captures the aggregate evolution of work effort through stocks, flows, and feedback loops representing quality management, rework, and approval processes. The second stage introduces the ABM component, which simulates decentralized decision-making, task execution, and learning at the agent level. These two modeling paradigms are then coupled mathematically through a bidirectional data exchange architecture that enables continuous interaction between individual task states and system-level indicators. This hybridization is theoretically motivated by the fact that, in complex systems, aggregate dynamics often emerge endogenously from microlevel interactions and cannot be adequately represented by purely aggregate or equilibrium-oriented models, particularly when execution is discrete, heterogeneous, and subject to coordination constraints.^21^

The subsequent sections detail each component and its role in the hybrid architecture. Section 2.1 presents the development of the SD model and its stock–flow formulation for R&D contexts. Section 2.2 introduces the ABM layer and explains its integration with the SD framework. Section 2.3 formalizes the coupling logic, showing how information is exchanged between layers through aggregation and feedback functions. Section 2.4 extends the model to estimate technological maturity using a probabilistic TRL formulation derived from simulation results. Section 2.5 describes the Simulation Process Manager (SPM), the integration engine that synchronizes discrete agent behavior with continuous SD dynamics. Finally, the concluding subsections address parametrization, validation, and the guiding hypotheses that underpin this first implementation of the hybrid framework. Together, these methodological steps provide a structured foundation for analyzing how microlevel task behavior, macro-level system feedback, and maturity progression coevolve within R&D projects.

### 2.1 System Dynamics Model

#### 2.1.1 System Dynamics: Quantitative and Qualitative Aspects

System dynamics is a methodological approach for analyzing the evolution of complex systems through endogenous feedback loops, stocks (accumulations), flows, and time delays.^20,22^ Its core strength lies in linking qualitative causal reasoning with quantitative simulation, enabling the representation of nonlinear behavior arising from interdependent processes. This makes SD particularly suitable for modeling R&D projects, which are characterized by uncertainty, iterative learning, and feedback-driven rework cycles.

In SD models, stocks represent accumulated system states, such as the remaining work, validated outputs, or available resources, while flows describe the rates at which these states change over time. The relationship between stocks and flows is expressed through integration, such that the value of a stock at time *t* results from the cumulative balance between its inflows and outflows

1 $$Stock\;\left(t\right)=\int_{t_{0}}^{t}\left[Inflow\;\left(s\right)-Outflow\;\left(s\right)\right]ds+Stock\;\left(t_{0}\right)$$

In discrete-time simulations, this formulation is approximated using finite time steps

2 $$Stock\;\left(t+\Delta t\right)=Stock\;\left(t\right)+\sum_{i}{Inflow}_{i}\left(t\right)\Delta t-\sum_{j}{Outflow}_{j}\left(t\right)\Delta t$$

Within R&D contexts, stock-flow structures are well suited to represent iterative execution and rework processes. Rework, rather than being treated solely as inefficiency, captures learning and uncertainty reduction mechanisms that are intrinsic to innovation activities. Feedback loops arising from quality assessment and review stages can either reinforce progress or introduce balancing effects that delay completion.

Causal Loop Diagrams (CLDs) complement this quantitative formulation by supporting the conceptual identification of reinforcing and balancing feedback mechanisms. CLDs are widely used during model conceptualization to structure expert knowledge and ensure internal consistency before formalization into stock–flow equations.^20^ Together, stock-flow models and CLDs provide a coherent framework for representing feedback-driven dynamics in R&D project execution.

Building on these principles, the present study adopts SD as the macro-level backbone of the proposed hybrid framework, providing a transparent and interpretable representation of aggregate project dynamics. The SD formulation is subsequently extended through direct coupling with an agent-based layer, as detailed in the following sections.

#### 2.1.2 A Novel Approach Based on System Dynamics for R&D

This study proposes a novel model that captures the system-level dynamics of R&D project execution through an SD submodel that simulates the continuous and cumulative evolution of work across key project stages. The formulation represents a simplified but representative structure of R&D workflows, reflecting the influence of rework, quality management, and approval processes on project progress and technological maturity. As detailed in Section 2.1.1, SD provides a robust foundation for modeling feedback-driven processes through differential equations, accumulations (stocks), and transition rates (flows).

The structure of the proposed SD model draws inspiration from the project planning framework presented by Shafieezadeh et al.,^6^ which simulates uncertainty and rework propagation in capital-intensive projects. That formulation incorporated structured work phases, deterministic review stages, and feedback-driven re-execution loops to represent project execution. The present model adapts these elements to R&D settings, extending them through direct interaction with an agent-based layer. Engineering management structures are simplified to reflect the inherent uncertainty and exploratory character of R&D, while deterministic interactions involving resources and infrastructure are maintained and captured through the ABM component, described in the next section.

Figure 1 presents the simplified stock-and-flow architecture of the SD model proposed in this work for R&D. Task effort progresses through four main stages (work to do, internal quality management, client review, and approved work). Each transition is governed by a flow equation that defines the rate at which effort moves from one stock to the next. Re-execution loops return part of the effort to previous stages when quality deficiencies or rejections occur, capturing the iterative feedback process characteristic of R&D activities.

**Figure 1:**
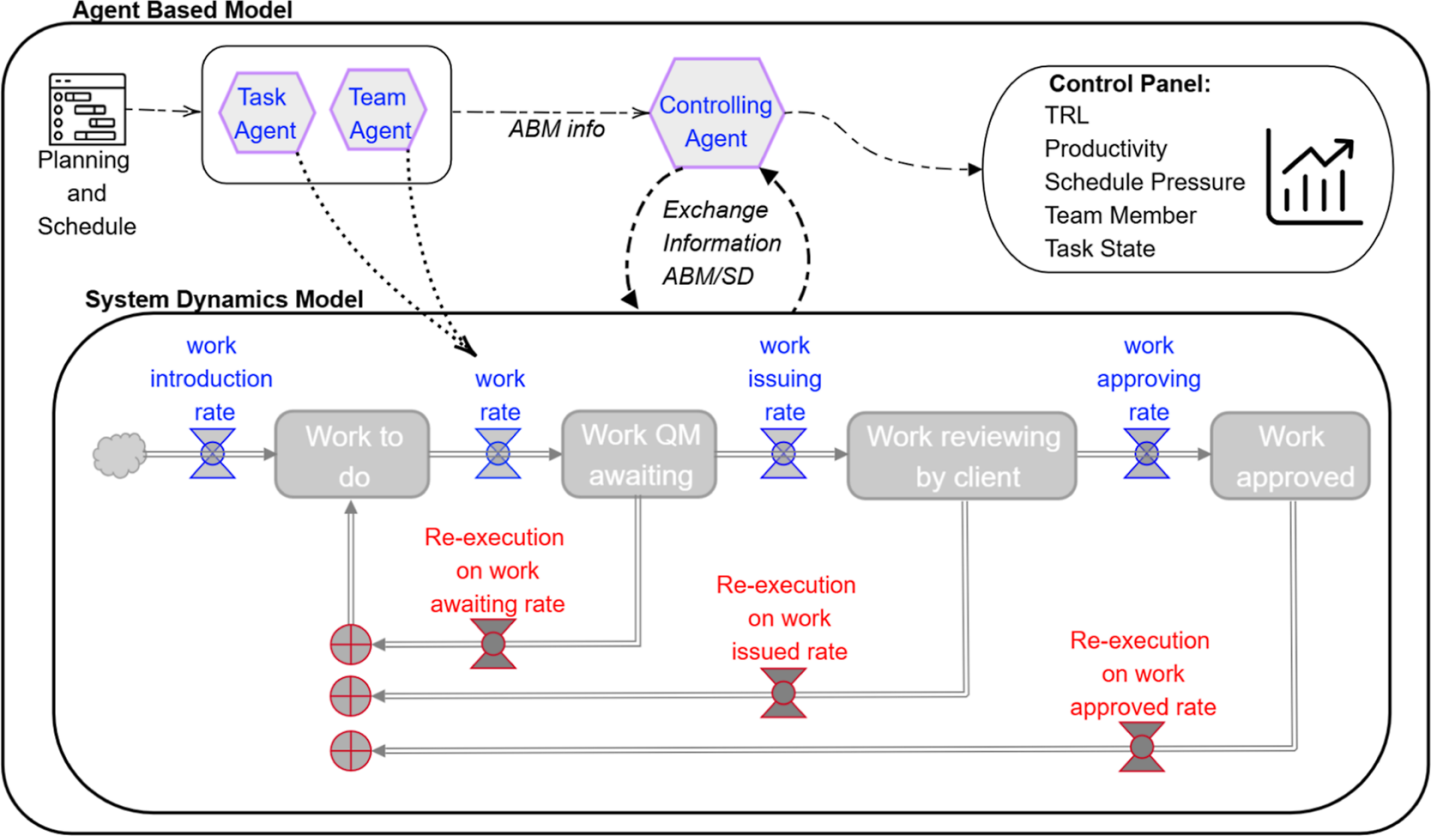
Simplified stock-and-flow diagram of the SD model. Rework is returned from multiple quality gates to the work to do stock via separate re-execution flows.

The SD submodel does not represent individual events or decisions but instead captures the evolution of aggregate quantities, such as available tasks, completed work, and review workloads over time. Differential equations define the rates of change (flows) between accumulated state variables (stocks), enabling the simulation of workload progression and rework cycles consistent with real-world project operations. Within the hybrid modeling framework, the SD model serves two primary functions: (i) aggregating microlevel outputs from the ABM into system-level indicators and (ii) providing a dynamic representation of overall project behavior to identify bottlenecks, rework dynamics, and schedule risks.

#### 2.1.3 Stock and Flow Structure

The SD submodel proposed in this work represents R&D project execution as a continuous flow of work evolving through successive development stages. The underlying architecture, shown in Figure 1, consists of interconnected stocks, flows, converters, and constants that describe the dynamic balance among completed, ongoing, and pending research activities.

In the context of R&D, stocks correspond to cumulative states of work or knowledge at specific stages of the project. As summarized in Table 1, four main stocks define the system:

- Work to do (WTD_*i*_) represents planned research activities or experimental tasks awaiting execution, analogous to the project’s backlog of scientific or engineering work.
- Work under quality assessment (WQA_*i*_) denotes ongoing verification and validation processes, such as data analysis, prototype testing, or internal peer review, which determine whether intermediate outputs meet technical requirements.
- Work under client review (WCR_*i*_) captures the stage where deliverables are externally assessed by stakeholders or funding agencies, equivalent to external evaluations, audits, or collaborative review meetings in R&D programmes.
- Work approved (WA_*i*_): represents completed and validated work that has successfully passed all internal and external assessments, contributing to technological progress and readiness.

**Table 1:** Stock Variables in the SD Model, Representing Cumulative Work at Key R&D Stages^a^

Stock	Description
WTD_*i*_	Work to do: accumulated R&D activities or experiments planned but not yet initiated, representing the project’s backlog of scientific or engineering tasks
WQA_*i*_	Work under quality assessment: ongoing research activities undergoing internal validation, data analysis, or prototype testing within the team
WCR_*i*_	Work under client review: deliverables or reports under external evaluation by funding agencies, partners, or industrial stakeholders
WA_*i*_	Work approved: completed and validated work that has successfully passed all internal and external reviews, contributing to project maturity and TRL progression

a Each stock is indexed by task *i*, preserving task-level resolution across the project execution. All variables are expressed in units of work (e.g., person-weeks).

Transitions between these stages are regulated through flows, as listed in Table 2. Forward flows, such as the work introduction rate (WIR_*i*_) and the work rate (WR_*i*_), control the initiation and progression of research activities. In contrast, re-execution flows (RE_*i*_) return a fraction of the workload to earlier stages when new findings, experimental inconsistencies, or approval rejections require additional work. This cyclical mechanism captures the iterative learning process typical of R&D, where uncertainty, discovery, and refinement occur simultaneously.

**Table 2:** Flow Variables in the SD Model, Representing Rates of Transition between R&D Stages^a^

Flow	Description
WIR_*i*_	Work introduction rate: inflow of new R&D tasks generated from project planning or agent-based inputs
WR_*i*_	Work rate: rate at which tasks progress from execution to internal quality assessment, influenced by team performance and workload
WIR_QM,*i*_	Quality management release rate: transition flow from internal validation to external review, reflecting deliverables ready for client evaluation
WAR_*i*_	Work approval rate: flow of tasks that complete all review stages and become finalized outputs contributing to TRL advancement
RE_QM,*i*_	Re-execution rate (quality management): portion of work returning to execution due to failed internal validation or test results
RE_C,*i*_	Re-execution rate (client review): work returned to previous stages after external rejection or requests for revision
RE_A,*i*_	Re-execution rate (approved work): minor rework or postvalidation adjustments prompted by new findings or process refinements

a Each flow is indexed by task *i*, preserving task-level resolution across the project structure. All variables are expressed in units of work per week.

Complementing these elements, Table 3 defines the auxiliary variables (converters) and constants that establish system-wide indicators. Converters include variables such as total work in process (WIP_*i*_), which aggregates ongoing effort across all stocks, and realized project duration (*D*_act_), which reflects the effective time required for project completion. Constants such as baseline duration (*D*_base_) and duration scaling factor (γ) define reference values and scaling relationships that modulate project timing under varying conditions of resource availability or complexity.

**Table 3:** Converters and Constants in the SD Model, Linking Accumulated Work and Temporal Performance in R&D Projects

Converter	Description	Unit	Domain
WIP_*i*_	Work in process: total active R&D effort aggregated across all project stages (sum of WTD_*i*_, WQA_*i*_, and WCR_*i*_), representing the total workload currently in execution or under review	Work units	[0, ∞)
*D*_act_	Actual project duration: cumulative time elapsed from the start of R&D activities to the completion of the last approved deliverable, reflecting the realized duration of the project	Weeks	[0, ∞)

Constant	Description	Unit	Domain
*D*_base_	Baseline duration: planned or reference duration for project execution, used as a benchmark to compare realized performance	Weeks	Fixed
γ	Duration scaling factor: proportionality constant that converts total effort into time, accounting for productivity variations or resource allocation levels	Work units/week	Fixed

All variables are indexed by task (*i*), preserving the task-level heterogeneity throughout the model. This indexing allows detailed tracking of progress across different R&D activities, ensuring that variations in scope, complexity, and rework intensity are explicitly represented. The units and valid domains of all variables are listed in Tables 1, 2, and 3.

The SD structure illustrated in Figure 1 is proposed here to be mathematically represented through the following system of differential equations, formulated according to the stock–flow relationship defined in eq 2.

3a $$\frac{dWTD_{i}}{dt}={WIR}_{i}-WR_{i}+RE_{QM,i}+RE_{c,i}+RE_{a,i}$$

3b $$\frac{dWQA_{i}}{dt}=WR_{i}-WIR_{QM,i}-RE_{QM,i}$$

3c $$\frac{dWCR_{i}}{dt}=WIR_{QM,i}-WAR_{i}-RE_{c,i}$$

3d $$\frac{dWA_{i}}{dt}=WAR_{i}-RE_{a,i}$$

From a systems perspective, the differential equations describe the dynamic balance of effort as it moves through successive R&D stages. The first equation governs the inflow of new tasks and the redistribution of effort due to rework, capturing how unresolved issues feed back into the initial workload. The second and third equations represent internal and external validation processes, where effort transitions between quality assessment and client review while accounting for possible feedback loops arising from failed evaluations. The final equation formalizes the accumulation of approved work, linking successful validation to the overall technological progress of the project. Together, these coupled equations define a structure in which work introduction, progression, validation, and rework continuously interact. This formulation allows the system to reproduce emergent behaviors such as learning cycles, schedule delays, and the nonlinear relationship between effort allocation and project maturity—key characteristics of real-world R&D processes.

As illustrated in Figure 2, each project task *i* is formally represented by an individual dynamic subsystem composed of a set of differential eqs 3a. These equations describe the temporal evolution of task-specific state variables, such as accumulated work, remaining workload, and completion status. The structures of the differential equations are identical across tasks, while their parameters and activation times depend on task dependencies and agent allocation. A task-specific dynamic model is enabled only when all of its predecessor tasks have been completed, ensuring that the system dynamics strictly follow the dependency structure depicted in the figure.

**Figure 2:**
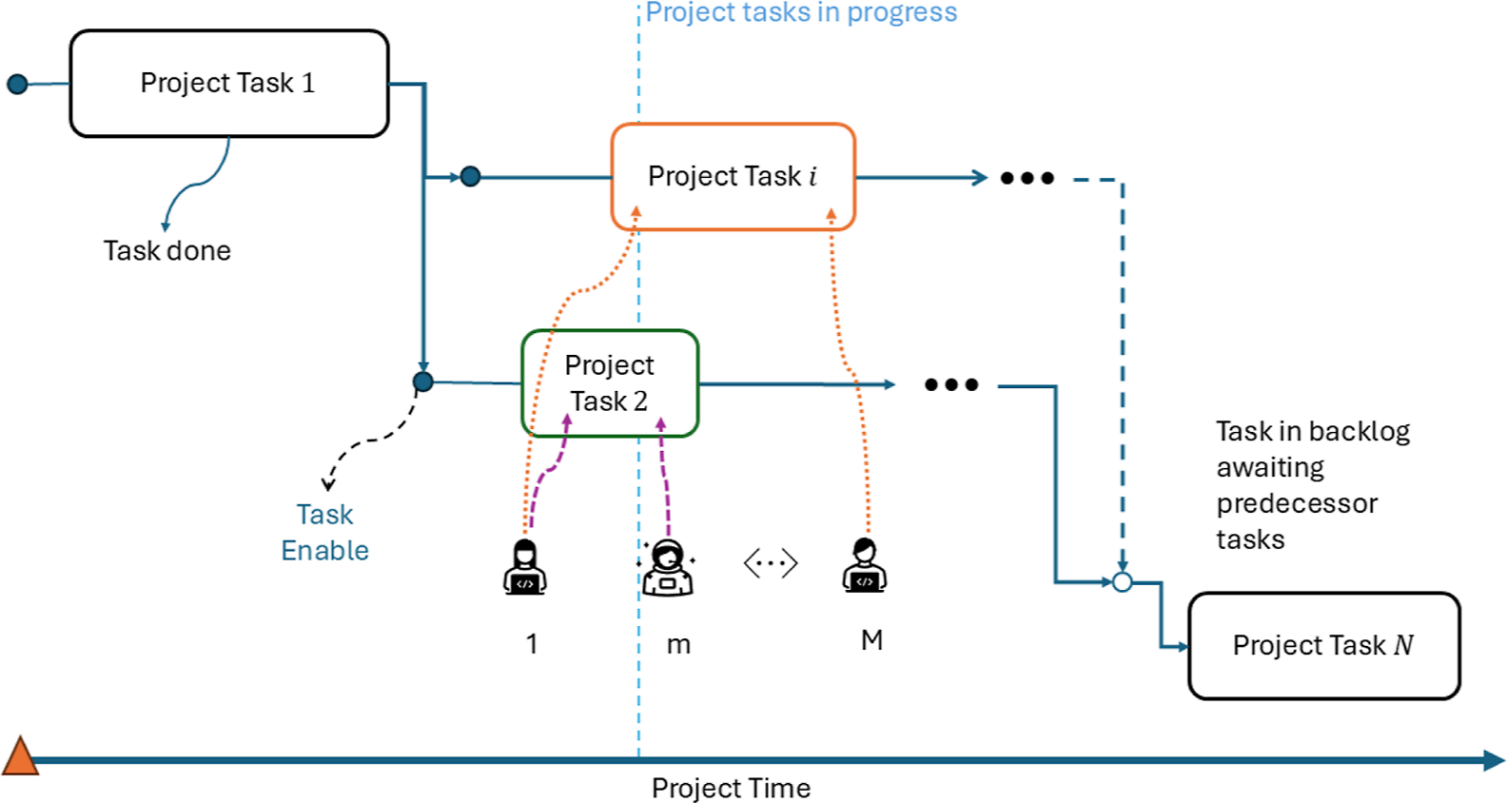
Schematic representation of task enabling and execution in the agent-based project model. Project tasks are organized according to precedence relationships; once prerequisite tasks are completed, subsequent tasks become enabled. Enabled tasks are executed by one or more team members selected from the available workforce, allowing parallel execution and heterogeneous allocation of agents over project time.

### 2.2 Hybrid Agent-Based Modeling for R&D

This work proposes a combined AB–SD approach designed for R&D projects. Both task level and project level are modeled in a single simulation environment. Individual or team behavior is represented by agents, while system-level mechanisms depict the project evolution. The architecture of the model is illustrated in Figure 3, which shows the exchange of information between system variables in the SD layer and agent behaviors in the ABM component.

**Figure 3:**
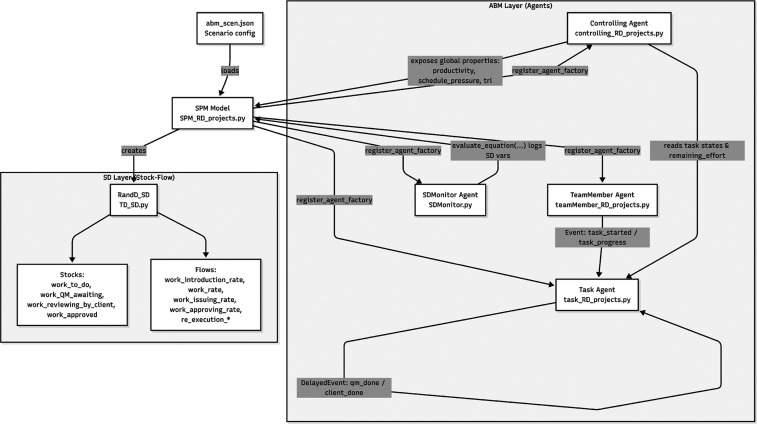
Architecture of the hybrid agent-based and system dynamics model. The ABM layer models task execution and team behavior through discrete agents, whose aggregated outcomes are transferred to the SD layer to drive stock–flow dynamics of work progression and rework. Macro-level indicators computed in the SD model are fed back to the ABM layer, influencing productivity and task execution and closing the micro–macro feedback loop.

The hybrid framework serves as a proof-of-concept platform for exploring execution dynamics of complex industrial R&D ecosystems such as offshore electrification and energy transition technologies. It enables systematic analysis of how microlevel mechanisms (task heterogeneity, learning-driven productivity, and iterative rework—collectively) shape macro-level outcomes, including schedule performance, resource utilization, and technological maturity under uncertainty and interdependence.

The ABM mechanism consists of three types of agents: a task, a team, and a controller. The task agent represents the evolution of individual tasks through a defined sequence of micro states characterized as open, in progress, internal quality review, client review, rework, and closed. While each task agent is assigned an initial effort, such an agent dynamically adjusts the required effort for completion. Additionally, these agents incorporate key properties, including task dependencies, rework probability, and the delay associated with task review.

The team member agent characterizes individual learning behavior, which is a key aspect influencing personal productivity. Furthermore, this agent manages the availability to admit new tasks. Ultimately, the controlling agent is responsible for assembling productivity and schedule pressure while also estimating the technological maturity and TRL evolution. These micro states are, then, defined as global properties that affect the SD flows. A formal description of the model’s logic, including the mathematical functions and rules that determine the agents’ dynamics, is provided in the next subsection.

### 2.3 Architecture and Coupling Logic

The previous subsections presented the SD and ABM components of the proposed hybrid framework, outlining their respective roles in representing the macro-level feedback structures and the microlevel behavioral dynamics of R&D projects. This subsection introduces the formal mathematical architecture that enables their integration. The objective is to define how information flows between the agent layer and the system-level model, establishing a coherent coupling logic that allows the microstate of individual agents to influence the aggregate variables of the SD component. The framework achieves this integration through an aggregation function that transforms the collective behavior of task agents into system-wide indicators, which are then used to update the SD equations governing workload, progress, and rework dynamics. Conversely, outputs from the SD layer inform the evolving states and decisions of the agents, creating a feedback loop between local actions and global system evolution. The following formulation describes this architecture and the underlying coupling mechanism that supports synchronization between discrete agent interactions and continuous system dynamics.

Let *A* denote the set of agents in the system, where each agent *a* ∈ *A* is characterized by state *x*_*a*_(*t*) in the overall state space *X* at time *t*. The collection of all agent states forms the system’s microstate, *X*_*t*_, and can be represented as

4 $$X_{t}=\left\{x_{a}\left(t\right):a\in A\right\}\subseteq X$$

An aggregation function is used to extract system-level indicators from the microstates to allow the ABM to influence the SD model. In order to couple the agent layer with the SD model, relevant properties from the task agent are aggregated into a set of macro-indicators, denoted as $S_{t}\in R^{n}$ . This aggregation is computed at each simulation step by the hybrid simulation environment, SPM, described in Section 2.5. The controlling agent operating within the ABM layer accesses these agent-level values and exposes them as global properties for intra-agent coordination. Specifically, it accumulates the remaining effort across all task agents in selected states. Formally, the aggregation function $V:P\left(X\right)\to R^{n}$ is defined as

5 $$S_{t}=V\left(X_{t}\right)={\left(\sum_{a\in A}w_{i}\left(x_{a}\left(t\right)\right)\right)}_{i=1...n}$$

where *w*_*i*_(*x*_*a*_(*t*)) are weighting functions that extract task-specific contributions to each aggregated macro-indicator, *S*_*t*_, based on the agent’s current state, *x*_*a*_(*t*). In the hybrid model, these functions are used to compute the total remaining effort in some of the task agent states”

6 $$\begin{array}{ll} w_{1}\left(x_{a}\left(t\right)\right) & =\{\begin{array}{ll} {remaining\text{\_}effort}_{a}\left(t\right), & \text{if}{\;x}_{a}\left(t\right)=“open”\\ 0, & otherwise \end{array}\\ w_{2}\left(x_{a}\left(t\right)\right) & =\{\begin{array}{ll} {remaining\text{\_}effort}_{a}\left(t\right), & \text{if}\;x_{a}\left(t\right)=“in\text{\_}progress”\\ 0, & otherwise \end{array}\\ w_{3}\left(x_{a}\left(t\right)\right) & =\{\begin{array}{ll} {remaining\text{\_}effort}_{a}\left(t\right), & \text{if}{\;x}_{a}\left(t\right)=“rework”\\ 0, & otherwise \end{array} \end{array}$$

The resulting macro-indicators, *S*_*t*_ are passed as exogenous inputs to the SD model at each time step, where they influence the flow rate of the SD stocks. For instance, the in.progress.effort affects the work rates, WR, while rework.effort affects the re-execution rates, RE. These flow rates then govern the stock variables, WTD, WQA, and WCR, which describe the system-level distribution of work in different phases.

In the opposite direction, the SD model produces system-wide performance metrics (e.g., schedule.pressure) based on the evolving state of its stock. These macro-indicators are exposed back to the ABM layer and can influence agent behavior. For example, increased schedule.pressure may lead to higher productivity or altered decision rules among Team Member agents. This feedback is operationalized through an update function

7 $$U:P\left(X\right)\times R^{n}\to P\left(X\right),,X_{t+1}=U\left(X_{t},S_{t}\right)$$

where *U* is the rule that determines how agent states evolve from one time step to the next, based on both the agent’s prior state and the current system-wide indicators.

Together, this feedback structure defines the core simulation logic of the hybrid model

8 $$X_{t+1}=U\left(X_{t},V\left(X_{t}\right)\right)$$

This recursive formulation establishes the dynamic coupling between microlevel agent states and macro-level SD. The model represents how individual task interactions collectively determine project-wide conditions and how these aggregated outcomes feedback to influence agent decisions in subsequent iterations. This bidirectional exchange forms a coevolutionary process in which local and global behaviors continuously shape one another. Through this structure, the hybrid framework enables a systematic examination of interdependencies and feedback mechanisms that characterize R&D project environments. It also provides a means to analyze how coordination strategies, workload propagation, and schedule pressure emerge from agent interactions and evolve under changing execution conditions, offering a foundation for exploring policy and management interventions in complex innovation systems.

#### 2.3.1 Behavioral Functions and Micro and Macro Feedbacks

Within the proposed hybrid framework integrating ABM and SD, learning, productivity, and schedule pressure are dynamically coupled through a closed feedback structure that governs task execution and project evolution. Agent behavior is defined through transparent, rule-based mechanisms rather than optimization or utility-maximizing assumptions. This formulation enables the emergence of coordination dynamics under uncertainty, while preserving model interpretability.

At the system level, schedule pressure is introduced as a global, dimensionless indicator representing the urgency to complete the remaining work, given the available time horizon and workforce capacity. It is formalized as

9 $$SP\left(t\right)=\min\left(\frac{E_{remaining}\left(t\right)}{t_{remaining}\left(t\right)\cdot N_{team}},SP_{\max}\right)$$

where *E*_remaining_(*t*) denotes the total remaining effort aggregated across all task agents in the open, in progress, and rework states, *t*_remaining_(*t*) is the remaining project time relative to the planned horizon, and *N*_team_ is the number of active team members. The upper bound SP_max_ prevents unrealistically large pressure values and is consistent with established project dynamics models that link urgency to cognitive and performance trade-offs.^20^

Schedule pressure influences execution dynamics through a nonlinear modulation of system-wide productivity. A global productivity factor is defined as

10 $$P_{global}\left(t\right)=f_{SP}\left(SP\left(t\right)\right)$$

where *f*_SP_(·) is a predefined lookup function. This formulation captures the empirically observed trade-off according to which moderate urgency enhances focus and performance, while excessive pressure leads to coordination breakdown, cognitive overload, and increased error rates.^23^

At the agent level, team members are modeled as homogeneous agents with identical behavioral logic for task selection and execution. At each simulation time step, available agents select eligible tasks. These tasks correspond to those in the open or rework states that are unassigned and whose dependency constraints have been resolved. Task selection follows a simple heuristic based on scenario-defined ordering. This abstraction prioritizes transparency and tractability while preserving the core coordination dynamics of R&D execution.

Individual learning is represented as an endogenous process driven by accumulated execution experience. Each agent’s personal productivity evolves according to a bounded sigmoid learning function

11 $$P_{personal,i}\left(t\right)=\frac{P_{\max}}{1+\exp\left(-\alpha n_{i}\left(t\right)\right)}$$

where *n*_*i*_(*t*) is the cumulative number of tasks completed by agent *i*, α is the learning rate parameter, and *P*_max_ denotes the maximum attainable individual productivity. This formulation captures rapid productivity gains during early task execution followed by diminishing returns as the experience saturates. These dynamics are consistent with established findings in innovation systems and learning-by-doing literature.^24,25^

The effective productivity applied by agent *i* to its assigned task results from the interaction between system-level conditions and individual learning effects. It is computed as

12 $$P_{eff,i}\left(t\right)=P_{global}\left(t\right)\cdot P_{personal,i}\left(t\right)$$

At each simulation time step Δ*t*, the corresponding reduction in remaining task effort is given by

13 $$\Delta e_{i}\left(t\right)=P_{eff,i}\left(t\right)\cdot \Delta t$$

This effort decrement advances task completion and triggers subsequent validation, rework, or closure events depending on task-specific outcomes.

Through this structure, microlevel learning and task execution directly affect the aggregate remaining effort, which in turn updates schedule pressure at the system level. The resulting pressure-dependent productivity feeds back to influence subsequent agent behavior and closes the feedback loop linking individual actions and system performance. This bidirectional coupling enables the emergence of nonlinear execution dynamics, including productivity saturation, rework amplification, and schedule-driven performance collapse, which are commonly observed in innovation-intensive and R&D project environments.^26^

### 2.4 Relating Project Execution to Cumulative Distribution of the Technology Redness Level

The hybrid AB–SD framework presented in the previous sections provides a dynamic representation of how R&D projects evolve through interconnected feedback and task-level interactions. While this structure captures the mechanisms driving project execution, it remains necessary to link these operational dynamics to a measurable indicator of technological maturity. This section introduces a probabilistic formulation that connects simulated project progress to TRLs, thereby extending the model’s capability to assess the likelihood that a technology will attain specific maturity thresholds over time.

Although the proposed hybrid model does not directly rely on empirical TRL milestone data, it enables the estimation of technological maturity through a statistical representation of cumulative project progress. The formulation establishes a probabilistic relationship between task completion and maturity attainment, allowing the interpretation of simulated project outcomes within a TRL-oriented framework.

Let *N*(*t*) denote the cumulative number of completed tasks at time *t*, and let *N*_total_ represent the total number of tasks within the project. A normalized measure of progress can then be defined as

14 $$x\left(t\right)=\frac{N\left(t\right)}{N_{total}}\in \left[0,1\right]$$

which serves as a proxy for technological advancement under the assumption that each completed task contributes incrementally to overall system maturity.

To represent the uncertainty and variability inherent in R&D execution, a cumulative distribution function (CDF) is derived from the logarithmic transformation of *x*(*t*). The distribution of ln­(*x*) is modeled through the Student’s *t*-distribution, which provides flexibility for limited sample sizes and captures the stochastic nature of progress trajectories. Following the approach of Kenley et al.,^15^ the cumulative probability of achieving a given maturity level is expressed as

15 $$F\left(x\right)=P\left(Maturity|x\right)=t_{\nu }\left(\frac{\ln\left(x\right)-\mathrm{\mu ̂}}{\sigma }\right)$$

where *t*_ν_(·) denotes the cumulative distribution function of the Student’s *t*-distribution with ν degrees of freedom. The parameters μ̂, *S*, and ν are estimated from multiple simulation runs *x*_k_^*^, each corresponding to a realization of normalized progress, and they are defined as follows

16 $$\mathrm{\mu ̂}=\frac{1}{n}\sum_{k=1}^{n}\ln\left(x_{k}^{*}\right)$$

17 $$\sigma =\sqrt{\frac{1}{n-1}\sum_{k=1}^{n}{\left(\ln\left(x_{k}^{*}\right)-\mathrm{\mu ̂}\right)}^{2}}$$

18 ν=n−1

Aggregating results from multiple stochastic simulation runs produces an empirical maturity profile that evolves with time. Additionally, the TRL evolution is modeled as follows

19 $$TRL={TRL}_{\min}+\frac{{TRL}_{\max}-{TRL}_{\min}}{1+\exp\left(-k\left(x-\mu_{TRL}\right)\right)}$$

This formulation does not replace formal TRL assessments but offers a quantitative approximation of the probability that a given technology achieves a specific readiness level, conditional on simulated execution dynamics. From a systems perspective, this probabilistic mapping bridges the operational layer of project execution with the strategic layer of technology assessment. It embeds TRL evolution within the hybrid simulation framework, transforming execution-based progress metrics into interpretable maturity indicators. The resulting maturity distribution supports decision-making under uncertainty, enabling project managers and policymakers to evaluate development trajectories, identify bottlenecks in technological advancement, and prioritize actions that accelerate readiness. In this way, the integration of TRL modeling completes the methodological framework introduced in this paper, linking task-level behavior, system-level feedback, and maturity evolution into a unified analytical approach for R&D management.

### 2.5 Integration Engine Role of the SPM

The hybrid AB–SD framework introduced in the previous section requires a coordinated mechanism to manage the interaction between its discrete and continuous components. This coordination is achieved through the Simulation Process Manager (SPM), which acts as the central integration engine of the model. The SPM governs the execution of simulation steps, synchronizes data exchange between the ABM and SD layers, and ensures temporal consistency throughout the simulation process.

Within the agent-based layer, the SPM instantiates and manages the lifecycle of the main agent classes—Task, Team Member, and Controlling agents. These agents collectively represent the decentralized decision-making and behavioral adaptation characteristic of R&D project execution, encompassing task progression, resource allocation, learning effects, and responses to schedule conditions. The SPM evaluates agent interactions and state transitions at each simulation step, maintaining coherence among interdependent activities.

Simultaneously, the SPM interfaces with the continuous-time SD model, which tracks aggregated project-level indicators such as total work in progress, quality assessment flows, and rework dynamics. Information generated within the ABM—such as the cumulative remaining effort, task completion rate, and rework demand—is aggregated and transferred to the SD layer as exogenous variables. The SD component, in turn, calculates system-wide indicators such as schedule.pressure and exposes them to the ABM, where they influence agent decision rules, productivity levels, and task prioritization.

The SPM therefore ensures a consistent feedback exchange between both modeling layers. Each simulation cycle follows a structured sequence: (i) agents update their states according to local conditions, (ii) aggregated variables are passed to the SD model, (iii) system-level feedback is computed, and (iv) the resulting indicators modify subsequent agent behavior. This bidirectional integration maintains logical consistency between micro- and macro-level dynamics, allowing the exploration of multiscale dependencies and emergent coordination patterns within R&D projects.

#### 2.5.1 Task Dynamics and Structural Dependencies

In the proposed hybrid AB–SD model, each R&D task is represented as an autonomous agent with fixed properties such as initial effort, rework probability, and predefined review delays. The initial attempt assigned to a task refers to the total amount of work required to complete it, expressed in abstract work units that capture the relative complexity, uncertainty, and resource intensity of each development activity, rather than real labor hours. These scenario-defined values draw from established concepts in staged technological development, including the TRL framework and the S-curve theory of innovation progression.^14,27,28^ Within this formulation, early-stage tasks are assumed to demand higher effort due to exploratory research, conceptual design, or experimentation, while later-stage tasks focus on refinement, integration, and validation. This reflects the common pattern in R&D processes, where uncertainty and learning intensity decline as the project moves toward maturity.

Task interdependencies are defined prior to simulation and remain static throughout execution. A task can only start once all of its designated predecessor tasks have been completed, establishing a directed dependency network. This structure allows for the controlled examination of systemic effects such as bottleneck formation, congestion in dependency chains, and the propagation of rework or delays through sequential phases. Although real-world R&D projects frequently exhibit evolving task relationships in response to discoveries or external constraints, the use of static dependencies simplifies analysis while preserving the essential causal feedbacks between interdependent development activities. This design enables the exploration of how structural properties—such as dependency depth or task clustering—influence overall project performance and technological advancement.

Each task progresses through a defined sequence of states that represent the core phases of the R&D execution cycle. After a task is initiated by a Team Member agent and executed to completion, it enters two successive validation stages: an internal quality management (QM) review and an external client review. These stages are modeled as deterministic delays with default durations of 1.0 and 2.5 weeks, respectively. This representation mirrors the structured review processes observed in R&D project management, where technical validation and stakeholder feedback function as essential control points in the innovation cycle. The deterministic formulation excludes stochastic variation, queuing effects, and resource constraints, which are outside the scope of this initial model but can be incorporated in future refinements.

This modeling choice is supported by the work of Shafieezadeh et al.,^6^ who demonstrated that explicit quality control stages in simulation models are essential for reproducing rework dynamics, performance feedback, and decision-making under uncertainty. In a similar manner, the review phases in the proposed model act as analytical checkpoints through which the effects of delay, rework, and information feedback can be observed. They enable investigation of how errors detected during validation influence schedule performance and workload redistribution across the project. While simplified, this formulation maintains interpretability and analytical transparency, providing a foundation for future extensions that incorporate stochastic review outcomes, concurrent verification processes, or variable resource capacities.

Tasks are modeled as heterogeneous, interdependent agents linked through structured review mechanisms. This formulation captures the iterative and feedback-driven nature of R&D execution and connects microlevel task behavior to the macro-level feedback processes simulated within the System Dynamics layer. The resulting integration supports investigation of how completion patterns, validation loops, and structural dependencies collectively shape project performance and the trajectory of technological maturity.

Finally, rework in the hybrid AB-SD model represents the need for task revision following inadequate outputs during internal or external review phases. At the agent level, rework is triggered probabilistically after a task has completed the client review. Each task agent is initialized with a predefined rework probability, typically ranging from 0.2 to 0.5. These probabilistic values determine the likelihood of being returned for re-execution. If triggered, the task transitions into a rework state, and a portion of its original effort is reassigned for completion. This abstraction is based on observed patterns in R&D and engineering projects, where tasks in earlier development stages are frequently more subject to adjustment due to technical immaturity than tasks in later stages. Literature on this demonstrates that structured review checkpoints and re-execution phases are critical mechanisms for managing uncertainty and detecting performance gaps.^6,19^

At the system dynamics layer, the cumulative remaining effort of all task agents in the rework stage is aggregated by the controlling agent and passed to the SD model as the variable abm.rework.effort. This aggregated indicator governs the re-execution flows within the SD model, as detailed in Section 2.3. The current implementation assumes that high-level effort measures sufficiently capture the magnitude of ongoing rework activity.

#### 2.5.2 Parameterization and Scenario Inputs

All simulation experiments are executed under predefined scenario configurations contained in a structured input file. This configuration defines fixed values for core parameters, including task attributes, agent decision rules, and system-wide constants. These parameters establish the boundary between the conceptual model and the simulation environment, ensuring consistency and reproducibility across experimental runs. The external configuration approach supports transparent hypothesis testing and comparative analysis while avoiding the inclusion of context-specific assumptions within the model structure itself. Scenario parameters can be adjusted without modifying the source code, facilitating sensitivity studies and systematic exploration of different R&D settings.

The integration of the ABM and SD layers represents the central feature of the hybrid framework. The coupling design follows the conceptual structure outlined by Howick et al.^29^ and is implemented through bidirectional data exchange summarized in Table 4. At each simulation step, agent-level task states and remaining efforts are aggregated into system-wide indicators that update the SD stocks and flows. In return, the SD layer produces global feedback variables, such as schedule.pressure, that influence agent productivity and task selection behavior. This reciprocal exchange maintains coherence between microlevel interactions and macro-level dynamics, allowing emergent behaviors to evolve consistently within the simulation.

**Table 4:** Biodirectional Integration Interfaces between ABM and SD Layers in the Hybrid Model

Interface	Description
Information flows from SD model to ABM model	
(1) Stock levels influence agent parameters	The SD model computes global project-level variables such as schedule pressure, which are exposed to all agents as external inputs. These values influence agent decision logic, such as how quickly assigned team members complete work
(2) Feedback propagation to agent performance	Fluctuations in SD stocks reflecting system-wide workload indirectly affect agent efficiency by modifying the shared productivity, simulating systemic schedule strain
Information flows from ABM model to SD model	
(3) Aggregated task state effort drives SD flows	The SD model consumes agent-level task states and remaining effort to compute aggregate values such as abm.in.progress.effort, abm.open.effort, and abm.rework.effort. These aggregated measures define the inflows and outflows in the SD stock-flow structure
(4) Behaviors of agents affect flows	Behaviors of agents in an ABM model can influence flows in an SD model by increasing/decreasing parameters used in equations for flows
(5) Agent task completions influence maturity dynamics	The cumulative number of closed task, tracked within the agent layer, informs the SD-level computation of technological maturity. This allows the model to translate microlevel execution into macro-level readiness assessments

The model operates as a proof-of-concept framework addressing a critical methodological gap in R&D modeling. Its objective is to demonstrate how a hybrid AB–SD approach can capture both execution dynamics and the probabilistic progression of technological maturity. The focus, therefore, lies in conceptual exploration and theoretical grounding. This approach aligns with the established role of system simulation as an exploratory method for studying complex socio-technical systems.^30^

Validation of the model focuses on three complementary dimensions: structural, behavioral, and hybrid consistency. Structural validation assesses whether the model’s internal logic aligns with the theoretical understanding of R&D projects. The consistency of the SD and ABM layers was verified through logic checks and parameter sensitivity tests to confirm expected directional behavior under varying conditions. Behavioral validation assesses whether simulation outputs reproduce recognized patterns of R&D system behavior, such as S-curve growth in technological maturity, delay propagation in sequential task chains, and performance constraints under limited resources. Hybrid-specific validation evaluates the correctness of interactions between the ABM and SD components, ensuring accurate data exchange and alignment of aggregated measures used for TRL estimation.^31^

Several factors constrained large-scale empirical verification, including the proprietary nature of detailed R&D project data, the nonstationary characteristics of innovation systems, and the limited access to industry experts within the current research scope. Despite these challenges, the model’s structure enables case-specific validation through expert judgment and historical comparison. This approach allows alignment of simulated patterns with expert understanding of R&D project dynamics and reference to documented historical cases, producing an expert-level empirical assessment of the model’s behavior. Such validation, while qualitative in nature, ensures that the simulation outcomes remain grounded in real-world experience and established knowledge. The results presented in the following section were obtained in order to confirm whether the hybrid AB–SD framework provides a consistent, transparent, and extensible foundation for continued refinement and domain-specific applications.

### 2.6 Model Hypotheses and Future Development Directions

The hybrid AB–SD framework presented in this work represents an initial step toward a formal and integrated modeling approach for analyzing the dynamics of R&D projects. Its purpose is to establish a foundational structure that connects individual task execution, team interactions, and system-level feedback mechanisms to technology maturity progression. While the model provides a consistent analytical foundation, its formulation incorporates a set of simplifying hypotheses designed to make the problem tractable and to prioritize conceptual clarity over empirical specificity. These hypotheses are summarized below, together with the reasoning that supports each choice:

- Homogeneous task contribution: Each task contributes proportionally to the cumulative technological maturity of the project. The proportion of completed tasks is treated as a proxy for readiness progression, assuming that all tasks have an equivalent marginal effect on the overall TRL evolution. Rationale: This simplification supports the first formalization of the coupling between task execution and TRL prediction. It preserves transparency and interpretability in the model’s initial version. Future developments can incorporate heterogeneous maturity weights based on task complexity, novelty, or criticality within the technological system.
- Simplified resource allocation: Team resources are represented through a continuous variable that modulates overall productivity. Rationale: This abstraction keeps the analysis centered on behavioral adaptation and system feedbacks, avoiding the computational burden of discrete scheduling or optimization algorithms. Later versions of the model can integrate adaptive allocation mechanisms or reinforcement learning strategies to simulate resource prioritization under uncertainty.
- Representative agent behavior: Agents follow rule-based decision logic that reproduces typical behavioral patterns in R&D teams—such as productivity adjustment, learning progression, and rework initiation—without modeling individual personality or cognitive variation. Rationale: This approach enables exploration of collective learning and coordination effects while maintaining computational tractability. Subsequent refinements may include more detailed behavioral rules or cognitive decision-making processes derived from empirical data.
- Aggregated feedback representation: System-level indicators, including schedule pressure, workload accumulation, and resource utilization, are computed as aggregate variables that apply uniformly across agents. Rationale: Aggregation ensures coherent information exchange between the ABM and SD layers, preserving the integrity of the coupling architecture. Future extensions could include network-based or hierarchical feedback to represent differentiated team structures or communication patterns.
- Idealized project topology: The R&D workflow follows a simplified linear progression through execution, internal review, client review, and approval stages, with feedback loops representing rework and learning. Rationale: This topology captures the cyclical and iterative essence of R&D without requiring project-specific workflow graphs. Future versions could incorporate parallel task streams, concurrent engineering structures, or dynamic task reconfiguration to capture the full complexity of industrial R&D projects.

These hypotheses define the scope of this first formulation and clarify that the model functions as an analytical tool for understanding R&D system behavior rather than a predictive simulator for specific industrial cases. The approach establishes a scalable base that can be progressively refined with empirical data, domain-specific parameters, and advanced behavioral rules, as demonstrated in the practical implementation of this work, which will be presented in the results section.

## 3 Results and Discussion

The following sections present the simulation experiment results obtained by using the hybrid AB-SD model introduced in Section 2. The primary aim is to assess the model’s internal behavior, sensitivity, and structural plausibility under varying conditions relevant to R&D project management.

### 3.1 Base Case

Given the limited availability of empirical data sets for early-stage R&D projects, the base scenario is designed as a representative reference experiment to examine the structural dynamics of innovation processes under controlled and transparent assumptions. This baseline configuration operationalizes the hybrid AB–SD model introduced in Section 2, decomposing the project into 15 discrete tasks distributed over a 156 week planning horizon. Each task is characterized by a specific workload and a probabilistic rework rate, capturing both technical uncertainty and iterative feedback processes that typically define high-uncertainty R&D environments.

In this configuration, tasks are executed in parallel without precedence constraints, forming a benchmark condition that isolates task-level variability and resource allocation effects from higher-order structural dependencies. This design serves as a reference model for subsequent comparative experiments, facilitating a systematic evaluation of how interdependencies, sequencing, and resource constraints impact system performance and technological progression.

Task complexity and uncertainty are stratified across the stages of technological maturity, following the TRL framework for the chemical industry proposed by^32^ As detailed in Table 5, tasks are organized into four TRL-based phases, spanning from conceptual ideation (TRL 1–3) to system-level validation and precommercial integration (TRL 8–9). Early innovation stages (Tasks 1–4) encompass high-effort, high-risk activities such as concept formulation and feasibility testing, reflected in the largest workloads (16 to 22 work units) and rework probabilities (0.42–0.50). Intermediate stages (Tasks 5–11) represent prototyping and validation phases, characterized by progressive uncertainty reduction. Final stages (Tasks 12–15) correspond to late-stage development, with minimal rework risk (0.10–0.18) and lower task effort (5–10 work units), consistent with increasing technological maturity and stabilization.

**Table 5:** Task Group Characteristics in the R&D Simulation Model

Task Number	Typical Tasks Covered	Effort Range [Work Units]	Rework Probability Range
1–4	Concept development, literature review, feasibility analysis	16–22	0.42–0.50
5–8	Prototype building, component validation	13–16	0.28–0.45
9–11	Demonstration activities, prototype testing	10–12	0.15–0.25
12–15	Integration, commercialization	5–10	0.10–0.18

The simulation operates at a temporal resolution of 1 week per time step. Agent properties governing team member behavior are specified to reflect generalized R&D learning curves and bounded productivity profiles. As detailed in Table 6, each agent begins with baseline productivity, incrementally increasing performance over time through a learning coefficient of 0.2, subject to a maximum output ceiling of 3.0 work units per week. Empirical and theoretical studies inform these parameter values of performance dynamics in knowledge-intensive project environments.

**Table 6:** Reference Values for Model’s Parameters

Property	Value
deadline	156 weeks
time step	1.0 weeks
team member	5.0
team.member.learning.factor	0.2
team.member.max.productivity	3.0

#### 3.1.1 Experiment 1—Dependencies

The first experimental scenario investigates the structural implications of task interdependencies on project execution dynamics within the hybrid AB–SD simulation framework. Drawing upon the modeling logic and assumptions about interdependencies introduced in Sections 2.6, the experiments contrast two extreme configurations: (i) a parallel task structure, representing the base-case scenario, and (ii) a strictly sequential configuration, in which all 15 project tasks are subject to linear precedence constraints.

In the parallel configuration, all tasks are assumed to be mutually independent, allowing simultaneous execution, provided that agent availability is met. This abstraction represents an idealized condition of maximum concurrency and absence of dependency-induced delays. In contrast, the sequential configuration requires each task to await completion of its predecessor, representing more constrained and risk-averse project logic often applied in high-dependency environments such as critical path scheduling. All other model parameters, including task attributes, agent behavior, simulation horizon, and performance coefficients, remain unchanged between configurations, as listed in Tables 5 and 6. This design isolates the causal effect of dependency structure, following SD best practices for testing endogenous relationships.^33^

Table 7 summarizes the resulting performance metrics. Under parallel execution, the system exhibits high resource concurrency, with an average of 1.2 tasks in progress and a peak queue length of 10 tasks. The average cycle time, defined as the elapsed time from task initiation to closure, reaches 47.9 weeks. Flow efficiency, reflecting the proportion of active work relative to total cycle time, is moderate at 48.1%. Resource utilization (23.7%) and throughput (0.2 tasks per week) remain stable, and the project is completed within 72 weeks, well ahead of the 156 week deadline, achieving 100% on-time delivery.

**Table 7:** Performance Metrics Comparison for Parallel (No Dependencies) and Sequential (Strict/with Dependencies) Task Execution Scenarios

Metrics	Parallel	Sequential
Average cycle time [week]	47.9	24.9
Flow efficiency [%]	48.1	91.2
Peak Queue [tasks]	10.0	2.0
Average queue [tasks]	1.5	0.1
Peak tasks in progress [tasks]	5.0	5.0
Avg tasks in progress [tasks]	1.4	0.9
Utilization [%]	23.7	17.0
Makespan [weeks]	88.0	208.0
Throughput [tasks/week]	0.2	0.1
On-time [%]	100.0	66.7
Late [%]	0.0	33.3

In contrast, the sequential configuration produces markedly different dynamics. The enforced dependency chain extends the project span to 171 weeks, reducing throughput to 0.09 tasks per week. Despite this, the average cycle time per task decreases to 25.6 weeks, and flow efficiency improves significantly to 91.2%. Queue lengths and resource contention diminish, but this comes at the cost of systemic inflexibility and temporal bottlenecking. On-time delivery falls to 66.7%, with the remaining 33.3% of tasks completed beyond the 156 week target.

Further differences emerge in task state occupancy, as summarized in Table 8. The cumulative task weeks spent in rework states decrease by 88% in the sequential case due to reduced compounding of concurrent rework feedback. Marginal reductions are also observed in client review and quality management delays. These findings are consistent with empirical observations reported in previous SD studies of project governance and feedback propagation.^19^ The results confirm that dependency-driven structures can reduce variability and rework propagation but at the expense of adaptability and overall throughput.

**Table 8:** Cumulative Task-Weeks in Rework, Client Review, and Quality Management Review States for Parallel and Sequential Task Execution Scenarios^a^

Task State	Parallel [Task-weeks]	Sequential [Task-weeks]	Change [%]
Rework	25.0	3.0	–88
Client review	80.0	72.0	–10
QM review	40.0	36.0	–10

a A task-week is one task occupying a given state for 1 week.

To evaluate how task dependency structures influence perceived technological maturity under uncertainty, an empirical CDF was derived from multiple stochastic simulation runs for both parallel and sequential task execution configurations. This probabilistic measure, visualized in Figure 5, reflects the cumulative likelihood that the system has achieved a given level of technological readiness by a specific point in time. The CDFs were computed by aggregating normalized progress trajectories from 100 runs and smoothing them to highlight general trends. In this context, normalized progress is defined as the cumulative fraction of completed tasks over the project horizon, which serves as a proxy for maturity. Although this proxy assumes a task contribution to system maturity, as described in Section 2.4, it enables a consistent and interpretable metric for comparative analysis of task dependency structures.

Figure 4 presents the temporal evolution of task states for the parallel and sequential configurations in Experiment 1. The graphs show how the number of tasks in each state changes over time and how this dynamic reflects differences in workflow coupling, concurrency, and completion patterns. In the parallel configuration (top plot), the number of open tasks decreases rapidly within the first 40 weeks as most activities are launched simultaneously. The in-progress curve initially rises steeply, reaching a peak corresponding to the full utilization of available resources, and then gradually declines as tasks are completed. The closed curve increases quickly and stabilizes well before the 156 week deadline, showing that all tasks are finalized within approximately half of the project duration. This pattern characterizes systems with high concurrency, short feedback loops, and greater exposure to rework caused by simultaneous development. Such oscillations between open and in-progress states replicate the empirical dynamics observed in flexible R&D programmes, where overlapping work packages accelerate output but generate coordination noise and iterative correction cycles.^19^ In the sequential configuration (bottom plot), the progression is more gradual. Only a few tasks are active at any given time, while others remain in the waiting dependencies state until their predecessors are completed. The closed curve increases stepwise, reflecting the gate-by-gate advancement typical of tightly coupled project networks. Completion occurs approximately 200 weeks after the start, exceeding the nominal 156 week deadline. This outcome reproduces the characteristic behavior of stage-gated industrial R&D systems, where dependency constraints mitigate rework and uncertainty but limit flexibility and increase overall duration.

**Figure 4:**
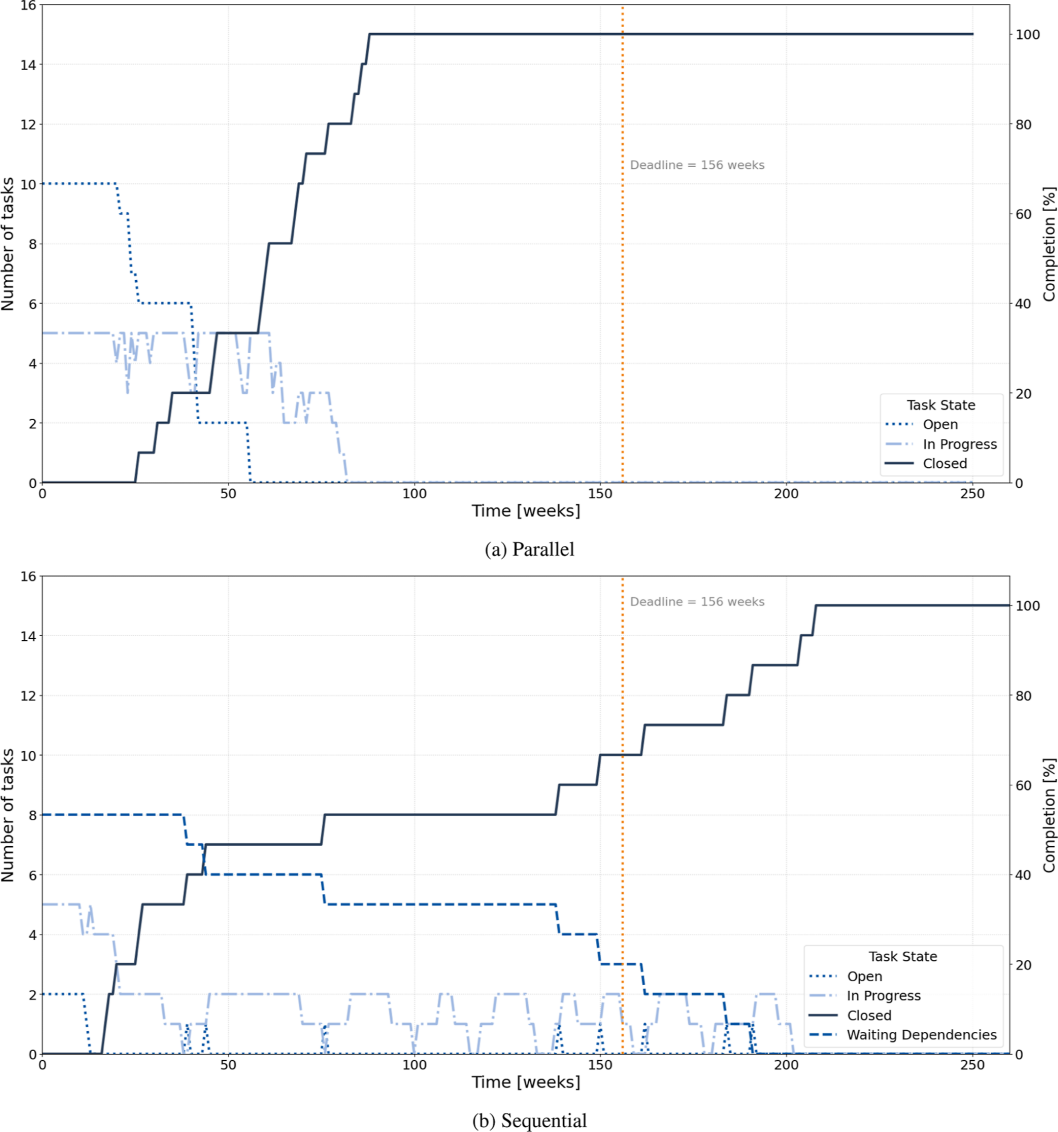
Evolution of task states over time for (a) parallel and (b) sequential configurations in Experiment 1.

The model therefore captures well-established empirical trade-offs documented in system dynamics and project-management research:

(i) Concurrency–rework relationship: high parallelism enhances early progress but amplifies iteration cycles.
(ii) Sequential stability: ordered execution improves predictability and quality assurance but extends the project duration.
(iii) Learning and coordination feedbacks: the damped oscillations and plateauing of completion rates reproduce typical learning-curve and workload-saturation effects observed in complex engineering and R&D settings.^20^

Overall, the simulation results demonstrate that the hybrid AB–SD framework accurately reproduces the temporal and structural patterns observed in empirical project studies. The evolution of task states, the timing of saturation, and the trade-offs between flexibility and control are consistent with observed dynamics in real R&D environments, confirming the model’s validity as a representation of technology-development workflows.

Figure 5 presents the cumulative distribution of technological maturity for the parallel and sequential configurations in Experiment 1. The parallel configuration exhibits a steeply rising maturity CDF, reaching a probability of approximately 90% by week 70 and plateauing near 1.0 at around week 90. This pattern reflects the high throughput and efficiency reported in Table 7 and demonstrates the advantages of full task concurrency. The steep gradient of the curve indicates limited interrun variability, consistent with low structural coupling and high schedule flexibility between tasks. The characteristic S-shape of the curve closely resembles classical logistic growth patterns used to describe Technology Readiness Level (TRL) progression in empirical studies.^14,27^ This similarity reinforces the conceptual validity of linking the simulated execution performance with probabilistic maturity assessments, even in the absence of explicit TRL milestone data.

**Figure 5:**
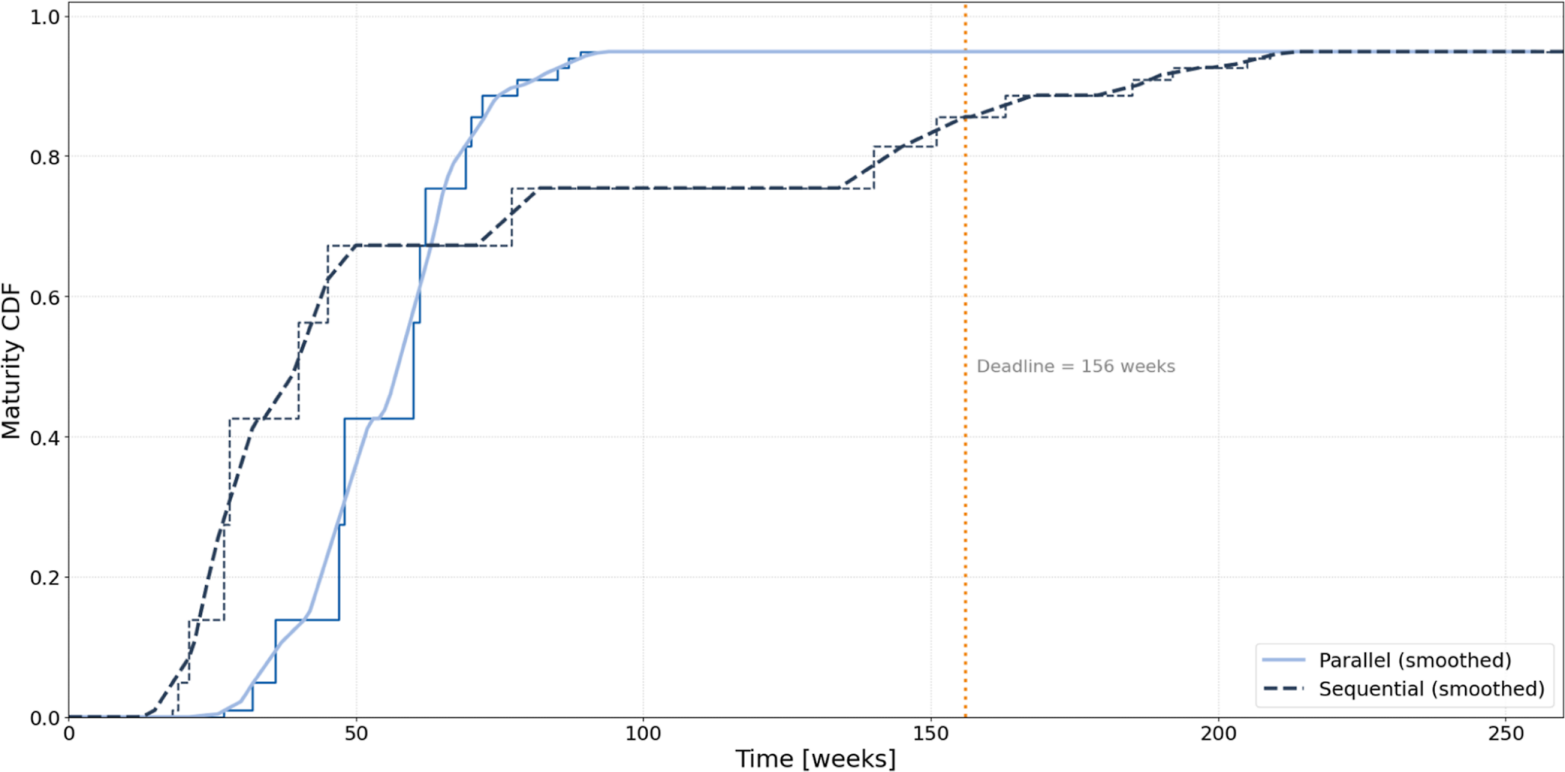
Cumulative distribution of technological maturity.

In contrast, the sequential configuration produces a slower and more extended maturity trajectory. The slope of the CDF is noticeably shallower, with the 90% probability threshold not reached within the 156 week deadline and instead attained only after week 200. This delayed progression reflects the restrictive effect of strict precedence constraints, where each task must await completion of its predecessor before initiation. The resulting dependency propagation introduces structural rigidity and greater temporal sensitivity, amplifying variability in cumulative completion across simulation runs. Consequently, the maturity CDF remains substantially lower within the planned time frame, signaling an elevated risk of underperformance and schedule overrun.

Overall, the comparison between the two curves illustrates how the project structure directly affects the dynamics of technological maturity. Parallel configurations accelerate early-stage learning and convergence toward complete maturity. In contrast, sequential configurations ensure control and quality but delay systemic progress—a trade-off widely recognized in the literature on SD and R&D project management.^19,33^

The simulation results from Experiment 1 provide strong evidence that the proposed model successfully reproduces how task-dependency structures fundamentally influence both operational dynamics and emergent maturity trajectories in R&D projects. While the performance indicators reported in Table 7 clearly distinguish the outcomes of parallel and sequential configurations, these results must be interpreted from a systemic perspective to reveal their broader implications for project behavior under uncertainty. From a system-dynamics standpoint, the emergence of consistent patterns across both task states and maturity CDFs validates the structural coherence of the hybrid AB–SD model. The parallel configuration demonstrates behavior characteristic of loosely coupled systems, marked by high concurrency, low systemic friction, and greater freedom of operation. These conditions yield a shorter makespan and higher throughput, accompanied by a sharply rising cumulative maturity trajectory that follows the classical S-curve pattern widely recognized in TRL and innovation management literature. The smooth, sigmoidal increase of the maturity CDF indicates that in unconstrained configurations, the stochasticity of individual task execution aggregates into a predictable, self-reinforcing progression of technological maturity. This outcome aligns with established SD theory, which posits that feedback structures with minimal coupling tend to stabilize over time through reinforcing resource utilization loops and adaptive learning effects. In this case, feedback on completed work accelerates subsequent progress by freeing up team capacity, reinforcing positive productivity–learning cycles, and sustaining high project momentum. Such behavior mirrors empirical observations in real R&D environments, where parallel experimentation and rapid iteration often lead to nonlinear yet stable growth in technological readiness.

Conversely, the sequential configuration introduces systemic constraints that fundamentally alter the emergent dynamics of the project. Although local performance indicators such as flow efficiency improve due to reduced rework, the overall project behavior becomes brittle and temporally extended. The delayed crossing of the maturity CDF beyond the critical threshold demonstrates how precedence constraints amplify propagation delays and diminish the system’s capacity to absorb variation. This rigidity limits adaptive responses and increases schedule sensitivity, revealing the inherent trade-off between control and agility within highly structured execution logics.

Furthermore, the probabilistic maturity CDF provides a valuable methodological perspective for interpreting the systemic consequences of execution structure under uncertainty. While the model does not explicitly include empirical TRL milestones, constructing the CDF from normalized task-completion trajectories enables the estimation of a probabilistic maturity envelope over time. Although this abstraction deviates from real-world R&D trajectories, it provides a meaningful approximation of how execution pathways impact the likelihood of achieving functional readiness within a defined planning horizon. The value of this approach lies not in predictive precision but in its ability to uncover how systemic characteristics—such as dependency structures, feedback loops, and rework propagation—shape the aggregate trajectory of innovation progress. In domains where timing is critical, such as compliance with regulatory milestones or stakeholder alignment, understanding how structural dynamics affect readiness probability can inform strategic scheduling, early-phase decision-making, and risk assessment.

It remains essential, however, to interpret these findings with appropriate epistemic caution. As outlined previously, the simulation framework is explicitly positioned as a proof-of-concept rather than a predictive or diagnostic instrument for operational use in industrial contexts. The model incorporates simplifying assumptions, most notably the exclusion of regulatory constraints and external shocks, as well as the use of synthetic parametrization, which collectively limit its external validity. The simulated project structure represents a generic abstraction that does not capture the specificities of electrification technologies or sectoral processes. Consequently, the results concerning task dependencies should not be interpreted as forecasts of actual R&D trajectories but as illustrative insights into how architectural choices influence innovation dynamics under high uncertainty.

Despite these limitations, the internal coherence observed between model structure, execution dynamics, and emergent maturity behavior provides confidence in the conceptual robustness of the approach. The hybrid AB–SD framework reproduces expected system patterns across different task configurations, generating plausible rework cycles, maturity lags, and agent-level bottlenecks without empirical calibration. This alignment between structural design and emergent behavior confirms that the model operates consistently within its intended scope and achieves its broader methodological purpose: to enable simulation-based exploration of the systemic interdependencies shaping complex R&D project dynamics.

The finding that sequential project execution reduces rework task weeks by 88% and slightly decreases both client and quality management review times confirms that the hybrid AB–SD model behaves as expected. This outcome aligns with established SD theory on rework feedback loops, which posits that high task concurrency, as observed in the parallel scenario, amplifies the propagation of defects. When multiple tasks overlap, errors tend to be detected downstream, triggering cascading rework cycles.^34^ In contrast, sequential execution constrains error propagation within each task cycle, thereby reducing the accumulation of hidden rework and contributing to more stable process control.

#### 3.1.2 Experiment 2—Effect of Team Size

The second experiment examined how team size influences project progress and task completion within the hybrid AB–SD simulation framework. This analysis was motivated by the hypothesis that human resource availability represents a critical leverage point in R&D project execution, particularly when combined with different task-dependency structures. The experiment was designed to isolate the effect of workforce capacity under controlled conditions and to evaluate how varying levels of team size modulate system behavior over time.

Two distinct task-execution logics were considered: (i) a parallel task-execution configuration and (ii) a sequential configuration, following the same structural setup described in Experiment 1. These configurations represent two archetypal operational modes in R&D workflows, capturing the contrast between concurrent and linearly dependent activities. The simulations were carried out for team sizes of one, two, three, four, five (base case), seven, and ten members. All other model parameters, as presented in Tables 5 and 6, were held constant across all experiments to ensure comparability. The underlying task structure, dependency matrix, and productivity-scaling rules were also kept unchanged.

The performance outcomes are summarized in Table 9, which reports total project duration, percentage of on-time task completion, and the rework ratio for each team size across both configurations. Figure 6 depicts cumulative task-completion trajectories for a representative subset of team sizes. A comparative visual summary of the project duration is also presented in Figure 7.

**Table 9:** Team Size Influence on Sequential Task Execution

	Parallel	Parallel	Parallel	Sequential	Sequential	Sequential
Team	Completion [Weeks]	On-time [%]	Rework Ratio [%]	Completion [Weeks]	On-time [%]	Rework Ratio [%]
1	377.0	23.08	2.3	318.00	60.00	1.79
2	333.0	66.7	0.08	310.00	73.33	0.15
3	220.0	93.3	1.13	N/A	N/A	0.33
4	88.0	100.0	0.24	350.00	53.33	0.29
5	88.0	100.0	1.24	264.00	66.67	0.19
7	80.0	100.0	0.17	N/A	N/A	0.15
10	89.0	100.0	0.12	N/A	N/A	0.12

**Figure 6:**
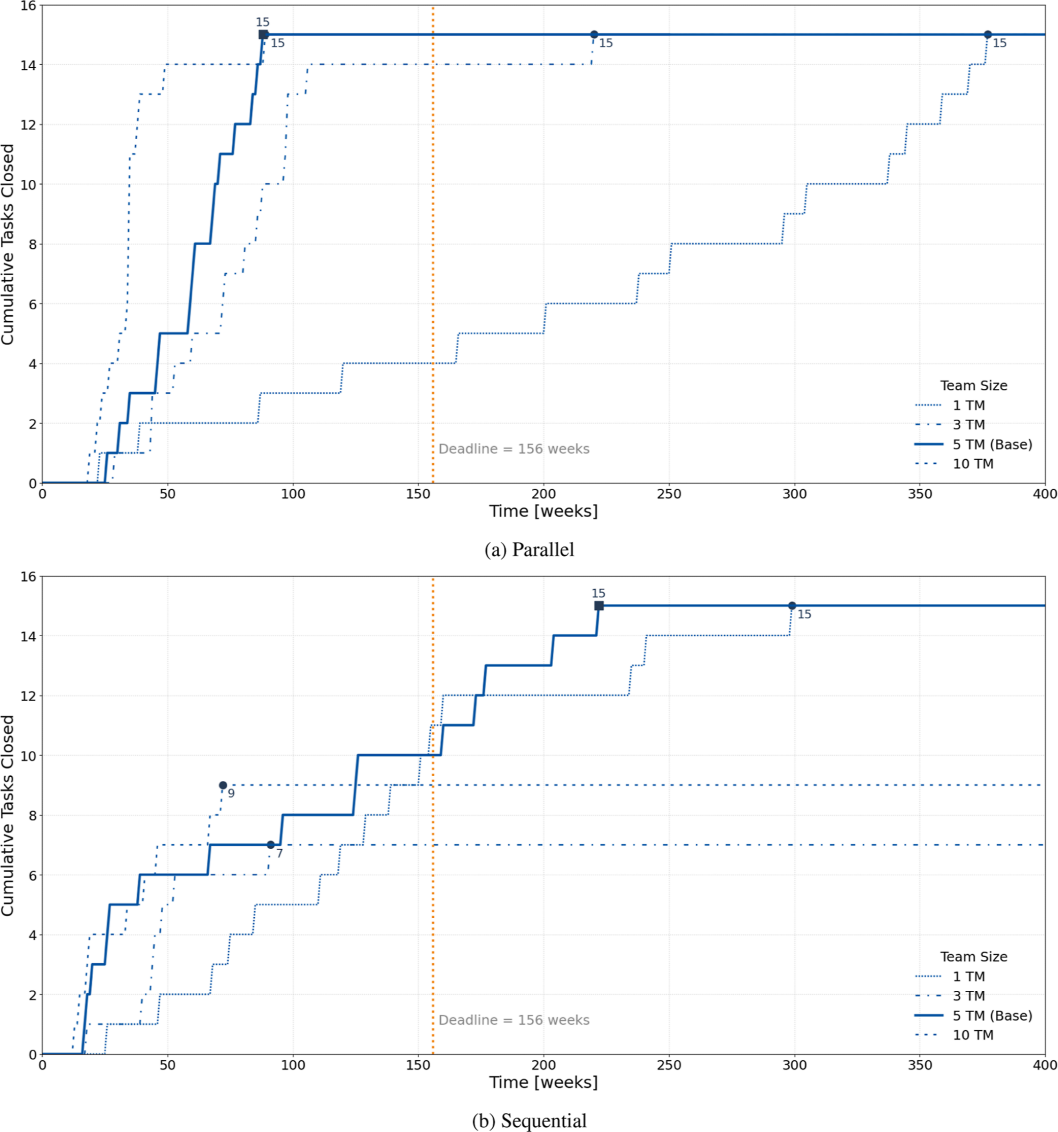
Cumulative number of closed tasks over time for selected team sizes under two execution configurations. (a) Parallel configuration with independent tasks executed concurrently and (b) sequential configuration, where tasks follow a fixed dependency order. The figure included a subset of team sizes to enhance visual clarity. The vertical orange line indicated the 156 week project deadline, and circle markers denote the point of full task completion.

**Figure 7:**
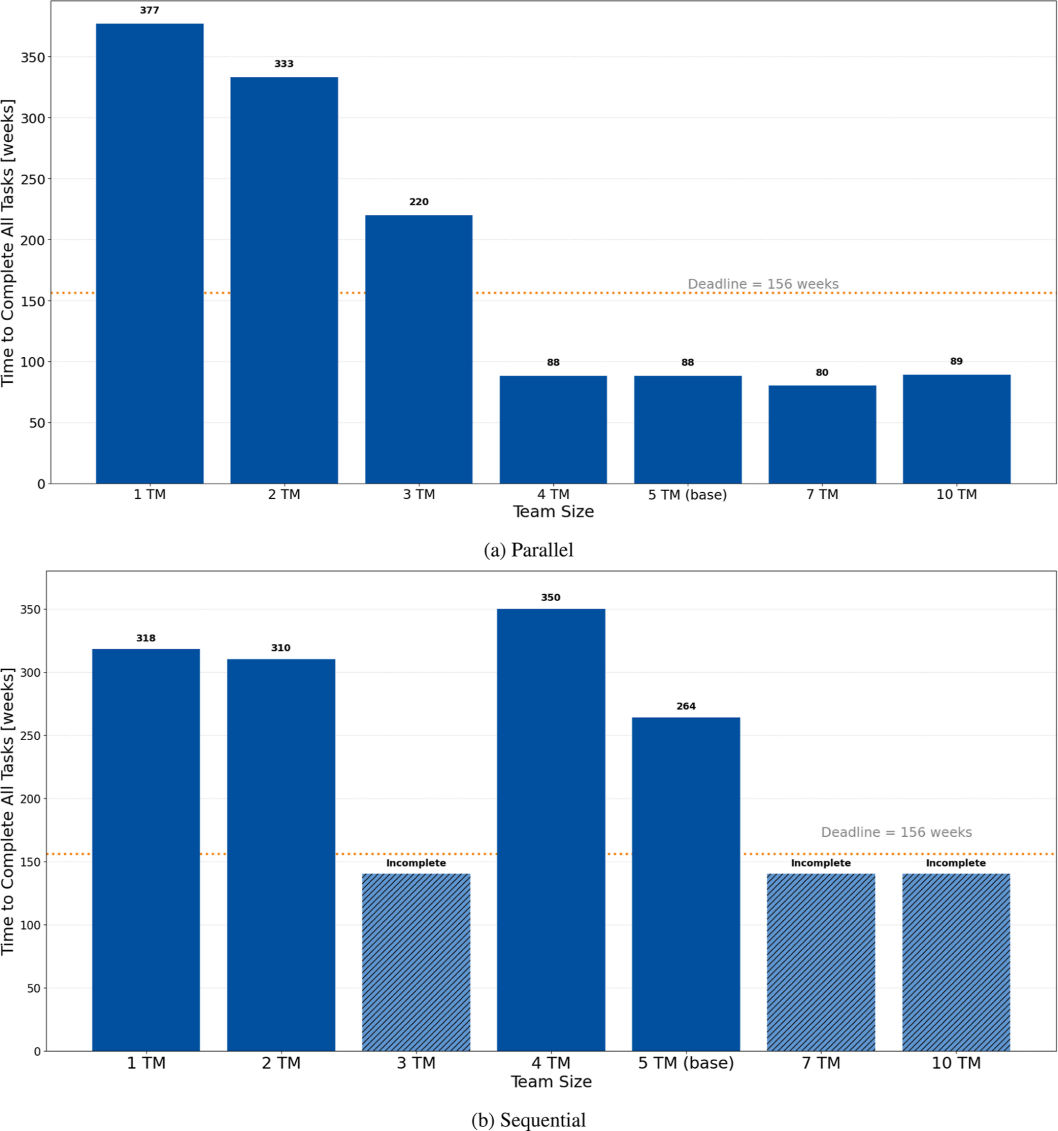
Total project duration for each time size under parallel (a) and sequential (b) task execution configurations. Bars represent the time required to complete all 15 tasks. Incomplete runs in the sequential case are shown with diagonally hatched bars, indicating that the project did not reach completion within the simulation window. The orange line marks the project deadline of 156 weeks.

In the parallel execution configuration, a clear and consistent relationship emerges between the team size and overall project performance. As team size increases, project duration decreases markedly, and on-time completion rates improve. For instance, a project executed by a single team member required 377 weeks to reach full completion, with only 23.1% of tasks delivered within the 156 week deadline. Once the team reached four members or more, the total project duration converged to approximately 88–89 weeks, achieving a 100% on-time completion rate. As shown in Figure 6a, increasing the number of team members significantly accelerates task completion, particularly during the early phases of execution.

In the sequential execution configuration, where tasks must be completed in strict order, the project performance varied substantially across different team sizes. Teams of one, two, four, and five members completed all 15 tasks within the 400 week simulation window. Among these, the five-member team achieved the shortest total project duration at 264 weeks, followed by the two-member team at 310 weeks, the one-member team at 318 weeks, and the four-member team at 350 weeks. Teams of three, seven, and ten members, by contrast, did not complete all tasks within the time frame, reaching only 7, 11, and 9 completed tasks, respectively. The bar charts in Figure 7b summarize these findings visually: solid bars represent fully completed projects, whereas diagonally hatched bars denote partial completion. None of the team sizes met the 156 week deadline, and only the five-membered team completed the entire sequence in under 300 weeks.

The results from Experiment 2 provide a detailed account of how team size influences project execution under both parallel and sequential task configurations within an R&D environment. In the parallel execution scenario (Figures 6 and 7a), increasing team size consistently reduced the total project duration and improved task completion rates within the 156 week deadline. A marked decrease was observed from 377 weeks for a single team member to approximately 88–89 weeks when team size reached four or more members. This behavior reveals an apparent threshold effect: a minimum team size of four to five is required to fully exploit parallelism, beyond which additional resources provide diminishing returns. This threshold aligns with the resource-saturation phenomenon described in the SD literature, where increasing resources initially enhances performance but eventually becomes constrained by coordination overheads and diminishing marginal gains.^20^ Although this result confirms the model’s sensitivity to workforce capacity, the parallel configuration should be interpreted as an idealized boundary condition rather than a realistic depiction of R&D operations.

It remains improbable that a five-member team could complete a complex offshore R&D project, spanning early-phase development to commercialization, within 156 weeks. This discrepancy suggests that the current parametrization may overestimate agent productivity, highlighting a key avenue for future model calibration using empirical data.

In contrast, the sequential execution scenario exhibited more complex and less intuitive dynamics. Teams of three, seven, and ten members were unable to complete all 15 tasks within the 400 week simulation window, reflecting inefficiencies arising from rigid task dependencies and allocation rules. These inefficiencies stem from the integer-based task distribution mechanism within the hybrid AB-SD model, as outlined in Section 2. Each team member sequentially claims tasks from an open queue and retains them through all stages—including quality management, client review, and potential rework—until completion. While this formulation realistically incorporates rework feedback loops, it also introduces scheduling bottlenecks when all agents are simultaneously occupied, preventing unassigned tasks from being initiated and causing premature stagnation in task progression.

These bottlenecks were particularly evident for teams of three, seven, and ten members, where uneven workload distribution resulted from the integer division of tasks among agents. For example, ten-member teams were assigned only one or two tasks per agent, leading to excessive waiting periods once the strict sequential dependencies were activated. Similar inefficiencies occurred in teams of three and seven, characterized by uneven workloads and extended idle times that ultimately prevented the initiation of final tasks. In all three cases, the alignment of task completion, review cycles, and agent availability led to situations where every agent remained occupied precisely when new tasks became available, thereby preventing their execution. This systematic undercompletion persisted even when the simulation horizon was extended to 600 weeks.

These findings align closely with insights from the SD and ABM literature, particularly the work of Shafieezadeh et al.,^6^ who demonstrated that larger teams do not necessarily yield proportional performance improvements in tightly coupled project environments. Increased coordination complexity and longer waiting periods frequently offset potential productivity gains under strong sequential dependencies. The incomplete task execution observed in the simulations is therefore a predictable consequence of the structural constraints embedded in the hybrid model rather than a stochastic anomaly.

Interestingly, this phenomenon did not occur in team configurations with one, two, four, or five members. These configurations naturally allowed at least one agent to become available at the precise moment when new tasks entered the queue, enabling continuous task progression and full project completion. This pattern suggests that the observed performance limitations primarily stem from discrete scheduling sensitivities rather than overall resource scarcity. The outcome underscores the crucial role of strategic task management in complex R&D systems, where even minor scheduling interactions can have significant systemic effects.

Although the hybrid AB–SD model effectively captures the intricate relationship between resource allocation, coordination, and task interdependency, it cannot yet be regarded as a definitive decision-making instrument. Nevertheless, the findings provide meaningful insights for R&D project management. They underline the importance of optimal team sizing, flexibility in task scheduling, and strategic coordination of dependencies. Implementing these practices can help mitigate the risks of delays and inefficiencies identified in the sequential-task configuration. While further model refinement and empirical validation are required to extend its predictive applicability, the current results already offer practical guidance for structuring team composition and managing interdependencies in complex R&D environments.

In the light of the results, future research should focus on expanding three key dimensions of the framework: (1) introducing heterogeneous task characteristics and nonlinear maturity relationships to better capture the uneven contributions of different R&D activities to technological progress, (2) integrating adaptive and learning-based decision mechanisms that enable agents to adjust behavior dynamically in response to changing project conditions, and (3) extending validation through empirical calibration with data from a larger variety of active R&D programs, aimed at refining parameter estimates, enhancing behavioral consistency, and increasing the framework’s predictive and explanatory depth. Overall, this formulation represents a first systematic attempt to link agent-level execution, system-level feedback, and TRL progression within a unified hybrid model. It provides a conceptual and computational foundation that can be extended to address the complexity of modern R&D environments and to support strategic decision-making in emerging technological fields.

## 4 Conclusions

The main goal of this study was to develop and demonstrate a hybrid modeling framework capable of representing the complex dynamics of R&D project execution and its relationship to technological maturity. The model integrates the continuous feedback representation of SD with the decentralized perspective of ABM, creating a unified structure for analyzing the interaction between task-level behavior, coordination mechanisms, and emergent project outcomes. This approach offers a methodological bridge between project execution dynamics and maturity assessment frameworks, providing a foundation for understanding how structural and behavioral factors jointly influence innovation progress.

The results confirm that the proposed framework reproduces the characteristic patterns of R&D system behavior, including variability in throughput, rework propagation, and review-cycle delays. These outcomes reflect known empirical trends and theoretical principles from SD, where the degree of task coupling governs the balance between flexibility, stability, and time-to-readiness. The experiments demonstrate that parallel configurations facilitate faster progress and higher throughput, albeit at the expense of increased rework. In contrast, sequential structures reduce rework but extend the overall project duration and limit adaptability.

A further contribution of this work is the introduction of a probabilistic representation of technological maturity based on cumulative distribution functions derived from simulated task completion. This feature enables a conceptual link between simulated execution and innovation metrics, such as TRLs. Although the framework does not rely on empirical TRL milestones, it produces maturity trajectories that capture the probabilistic evolution of readiness under uncertainty, allowing the early identification of potential risks and delays.

Within its proof-of-concept scope, the hybrid AB–SD model demonstrates internal coherence and conceptual robustness. The consistency observed between structure, behavior, and emergent maturity trajectories indicates that the framework functions reliably within its defined boundaries. It serves as an exploratory and decision-support tool that facilitates the examination of alternative project architectures, investment priorities, and schedule strategies in R&D environments. The overall contribution lies in establishing a transparent, extensible, and quantitative basis for simulation-based analysis of technological innovation, supporting the transition toward more data-informed and adaptive management of digital and industrial transformation processes.

## Supplementary Material



## Notes

Description of the python-based implementation of the hybrid ABM–SD framework and code availability; detailed specifications of task, team member, and controlling agents, including states and properties; agent attributes and system variables with corresponding units; additional experimental results assessing the effect of team size under parallel and sequential task structures; productivity, schedule pressure, rework dynamics, cumulative task completion, and maturity trajectories; and comparison of Technology Readiness Level (TRL) definitions (PDF)
