# Supplementary material for: An Agent-Based Modeling Dynamic Hybrid Model for Project Management in Research and Development
Source: Ind Eng Chem Res. 2026 Feb 26;65(9):5259–78. doi: 10.1021/acs.iecr.5c04351 (PMC12983320; doi:10.1021/acs.iecr.5c04351)
Supplement: Supplementary file 1 [file ie5c04351_si_001.pdf]

Supporting Information for Publication for: An Agent-Based Modelling Dynamic Hybrid Model for Project Management in Research and Development

Ind. Eng. Chem. Res.

Robson Wilson Silva Pessoa[a], Marie Hahn Naess[a], Julia Carolina Bijos[a], Carine Menezes Rebello[a], Danilo Colombo[b], Leizer Schnitman[c] and Idelfonso B. R. Nogueira[a,*]

[a]Department of Chemical Engineering, Norwegian University of Science and Technology, Trondheim 793101, Norway
[b]CENPES, Petrobras R&D Center, Av. Horácio Macedo 950, Cidade Universitária, Ilha do Fundão, Salvador 21941-915, Brazil
[c]Postgraduate Program in Mechatronics, Federal University of Bahia, Polytechnic School, R. Prof. Aristídes Novis 2, Federação, Salvador 40210-630, Brazil

[*]Corresponding author

A Software for Modeling

The hybrid ABM–SD simulation model proposed in this paper was implemented in Python. The implementation relies on the Business Prototyping Toolkit for Python (BPTK_Py), an open-source framework that supports the integrated simulation of system dynamics and agent-based models within a unified execution loop [1, 2]. The modular structure of the framework facilitated the implementation of task-level dynamic subsystems and agent-driven execution logic.

A.1 Implementation and Code Availability

The full source code, including the model structure, agent definitions, and simulation scripts, is publicly available at:

`https://github.com/ABM-SD/abm-sd-hybrid-framework`

The repository provides a reference implementation of the task enabling mechanism and the task-level dynamic subsystems described in Figure 2 and stock equations in the main text.

A.2 Conceptual Modeling Tool

BPTK_Py enables the integration of agent-based and system dynamics paradigms, allowing the co-evolution of micro-level task execution and macro-level workload dynamics. The framework provides abstractions for agent behavior, event-driven logic, and differential equation–based stock-and-flow structures, as well as built-in support for time-series data collection and post-processing using Python's scientific computing ecosystem.

B Agent-Based Model Design

The agent-based component of the hybrid AB-SD model represents the micro-level structure of an R&D project. The model comprises three primary agent types, *Task Agent*, *Team-Member Agent*, and a *Controlling Agent*. These agents interact dynamically to simulate task execution, personnel assignment, learning effects, and project-wide coordination. The agent structure and interaction logic are inspired by modeling patterns demonstrated in the BPTK_Py documentation by Transentis Labs [2]. While adapted and extended for the context of offshore R&D projects, this provided a valuable foundation for structuring the task–developer dynamics within the ABM framework.

B.1 Task Agent

In the agent-based layer of the hybrid model, *Task Agents* are used to represent individual R&D tasks. Unlike SD approaches, where such tasks might be defined via index-based stock arrays (e.g., in tools like Vensim), the ABM approach allows explicit modeling of task heterogeneity and dynamic interactions without requiring expensive SD simulation software or complex vectorization. To represent the diverse and interdependent nature of R&D activities, the simulation deploys multiple instances of the task agent, where each instance corresponds to a unique task characterized by its effort, dependencies, and execution dynamics.

Each task follows a defined lifecycle through a series of discrete states. A task agent is initially created in the `open` state but remains inactive until all its predecessors are resolved. If dependencies are present and not yet fulfilled, the task enters a temporary queue state, `waiting_dependencies`. Once all prerequisites are satisfied, the task becomes eligible for assignment and, upon being picked up by a team member, transitions into `in_progress`.

Work on the task proceeds according to the effort input the assigned team member provides. Upon completing the required workload, the task proceeds to an internal quality assurance review, `qm_review`, followed by an external client validation phase, `client_review`. Based on a probabilistic outcome, the task may either be approved and closed or returned for partial re-execution in the `rework` state. In such cases, a portion of the original workload is reassigned, simulating the real-world cycles that often occur during early-stage technology development of R&D projects.

A complete overview of the task agent's states and properties in the ABM framework is provided in Table S1, which outlines the variables that govern each task agent's behavior and lifecycle. The underlying assumptions and parameterization strategies for these variables are further described in Section Model Hypotheses and Future Development Directions.

Table S1: Underlying state and properties for the Task Agent in the ABM model structure.

Attribute	Description	Unit
State		
`open`	Task is ready to be picked up but work has not yet started	[-]
`waiting.dependencies`	Task cannot start until all predecessor tasks have been completed	[-]
`in.progress`	A developer is actively working on the task	[-]
`qm.review`	Task is undergoing internal quality management review	[-]
`client.review`	Task is being reviewed by a client or project lead for external validation	[-]
`rework`	Task failed client or quality review and requires partial re-execution	[-]
`closed`	Task has passed all reviews and is fully completed	[-]
Properties		
`effort`	Initial total workload required to complete task	[Work units]
`remaining.effort`	Remaining work left to complete the task	[Work units]
`dependencies`	List of task IDs, list of predecessor task IDs	[-]
rework probability	Probability that the task will require rework	[%]
`qm.delay`	Duration of the internal quality assessment process	[Weeks]
`client.delay`	Duration of the external client review process	[Weeks]
`assigned`	Boolean indicating whether the task is currently assigned to a team-member (true/false)	[-]

B.2 Team Member Agent

The *Team Member* agent represents an individual developer or engineer within the R&D project. Each agent operates autonomously, selecting tasks, performing technical and research work, and learning from experience/improving its productivity through cumulative learning, a process that reflects the acquisition of domain knowledge and task-specific competencies over time.

At any given point, a team member is in one of two states: `available` or `busy`. When available, the agent scans all task instances of the task agent for eligible assignments, specifically those in `open` or `rework` states that are not currently assigned and whose dependencies are fully resolved. Upon assignment, the team member transitions to the `busy` state and initiates task execution by triggering a `task.started` event in the corresponding task agent (see Table S2).

Work is performed incrementally during each simulation timestep, and the amount of effort contributed is governed by the agent's effective productivity, defined as the product of a global productivity factor and the agent's individual productivity level. At the same time, the global productivity factor is intended to reflect the system-wide effects, such as schedule pressure and workload intensity derived from the SD layer. Individual productivity evolves dynamically during the simulation. It is modelled using an S-curve learning function, which models the principle of experience-driven capability growth. Productivity increases as the agent completes more tasks, but follows a diminishing returns curve, capturing the realistic ceiling in performance improvement.

Table S2: Underlying state and properties for the *Team Member* agent in the ABM model structure.

Attribute	Description	Unit
State		
`available`	Team member is idle and ready to take on a new task	[-]
`busy`	Team member is currently working on an assigned task	[-]
Properties		
`task`	Reference to the currently assigned task, or `None` if unassigned	[Work units]
`completed.task`	Total number of tasks completed by the agent	[Count]
`learning.factor`	Sensitivity of productivity growth to task completion	[-]
`max.productivity`	Maximum personal productivity the agent can achieve	[-]
`personal.productivity`	Current productivity level based on cumulative learning	[-]

B.3 Controlling Agent

The *Controlling* agent is a centralized coordination mechanism in the hybrid model, acting as the interface between the ABM and the SD layer. Its primary function is to monitor and expose key global parameters, specifically `productivity` and `schedule.pressure`. These parameters influence agent behavior across the model. Additionally, it accumulates task-level workload data for synchronization with the SD model and could track the TRL trajectory for the R&D project in a more extended implementation.

Upon initialization, the agent sets baseline values for `productivity` and `schedule.pressure`, which conceptually represent the system-wide dynamics. The variables are registered as agent properties using the simulation engine's property system, enabling them to be accessed globally by other agents (e.g., team members) during simulation execution.

In addition, the *Controlling* agent aggregates task-level effort data. Specifically, it computes the total remaining effort across all *Task* agents. These aggregates are passed to the SD model as inputs (`abm.open.effort`, `abm.in.progress.effort`, and `abm.rework.effort`). This setup allows the SD to adapt in real time to shifts in task distributions.

A further key functionality of the *Controlling* agent is the dynamic estimation of technological maturity through a cumulative distribution function (CDF). The mathematical formulation and interpretation of the CDF are described in detail in Section 2.4. This CDF-based estimation support the evaluation of technological progress over time and offers an interpretable maturity index.

C Experiment 2 - Effect of Team Size on Project Execution

The tables S3, S4 and S5 summarize the results of Experiments 2.

The list below outlines how the 15 project tasks were distributed among agents for each team size. The allocation followed an even distribution approach, with any remainder tasks assigned to a subset of agents.

- For a team of 1 member, all 15 tasks are assigned to the single agent.
- For a team of 2 members, each agent is assigned 7 tasks, with one agent receiving an additional task (8 tasks).
- For a team of 3 members, each agent is assigned exactly 5 tasks.
- For a team of 4 members, three agents are assigned 4 tasks each, while one agent receives 3 tasks.

Table S3: Team size influence on parallel task execution

Team Size	Completion Time [Weeks]	On-time Completion [%]	Flow Efficiency [%]
1	377.0	26.7	10.7
2	333.0	66.7	60.6
3	220.0	93.3	33.9
4	88.0	100.0	36.4
5	88.0	100.0	48.1
7	80.0	100.0	59.5
10	89.0	100.0	79.1

Table S4: Team size influence on sequential task execution

Team Size	Completion Time [Weeks]	On-time Completion [%]	Flow Efficiency [%]
1	299.0	73.3	10.7
2	329.0	60.0	60.6
3	91.0	100.0	33.9
4	341.0	53.3	36.4
5	222.0	66.7	48.1
7	123.0	100.0	59.5
10	72.0	100.0	79.1

Table S5: Performance's metric.

Metric (SD)	Parallel	Sequential	Unit
WTD_{avg}	465.22	39.40	Work Units
WQA_{avg}	7.29	15.95	Work Units
WCR_{avg}	211.16	810.72	Work Units
WA	138.06	163.30	Work Units
WIR_{tot}	352.00	72.00	Work Units/Week
WR_{tot}	268.43	249.80	Work Units/Week
Rework Ratio	121.93	44.15	%

- For a team of 5 members, each agent is assigned exactly 3 tasks.
- For a team of 7 members, six agents are assigned 2 tasks each, and one agent receives 3 tasks.
- For a team of 10 members, five agents are assigned 2 tasks each, and five agents receive 1 task each.

D TRL

This information is available free of charge via the Internet at http://pubs.acs.org/.

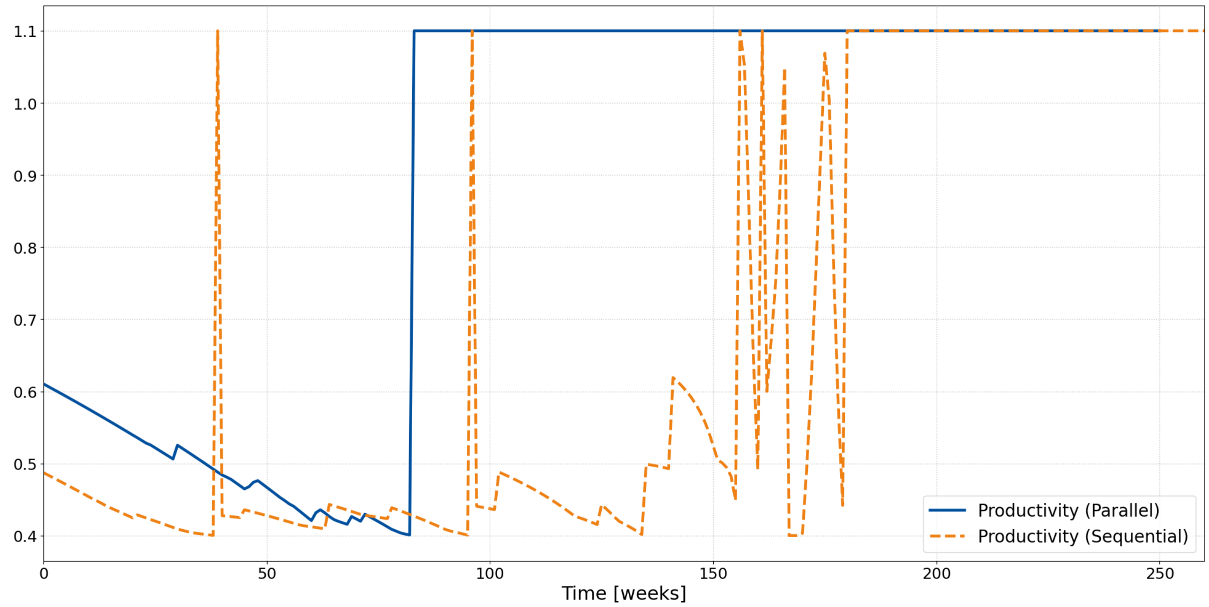

(a) Productivity for parallel (blue) and sequential (orange) configurations.

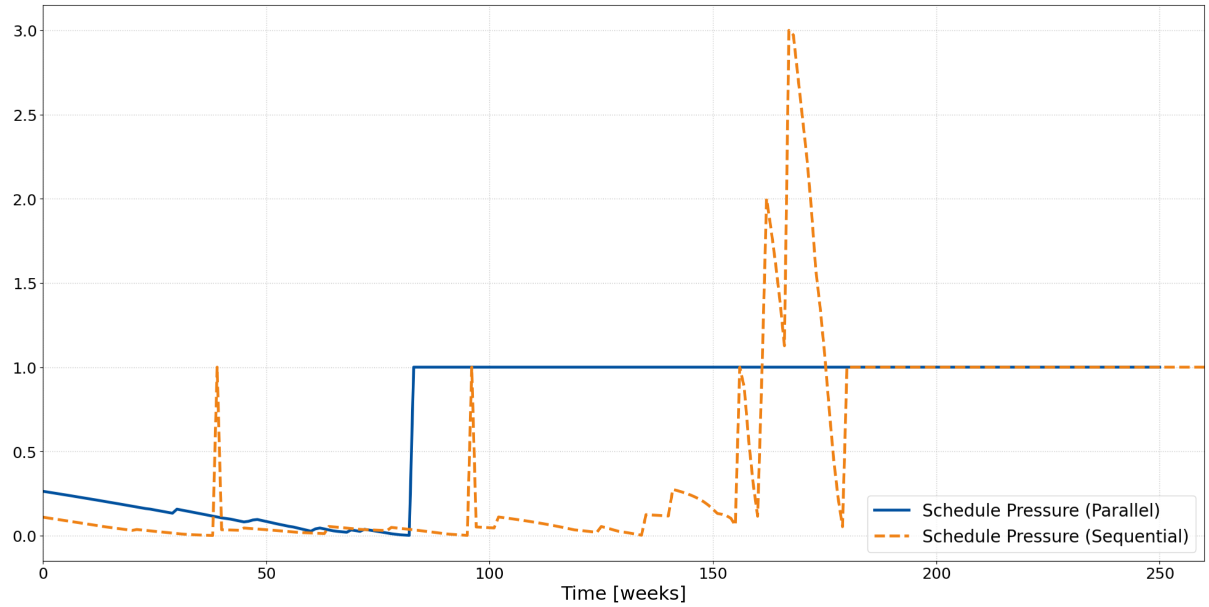

(b) Schedule pressure for parallel (blue) and sequential (orange) configurations.

Figure S1: Performance metrics for Experiment 1, two configurations of dependency: parallel and sequential. **(a)** displays productivity and **(b)** represents schedule pressure.

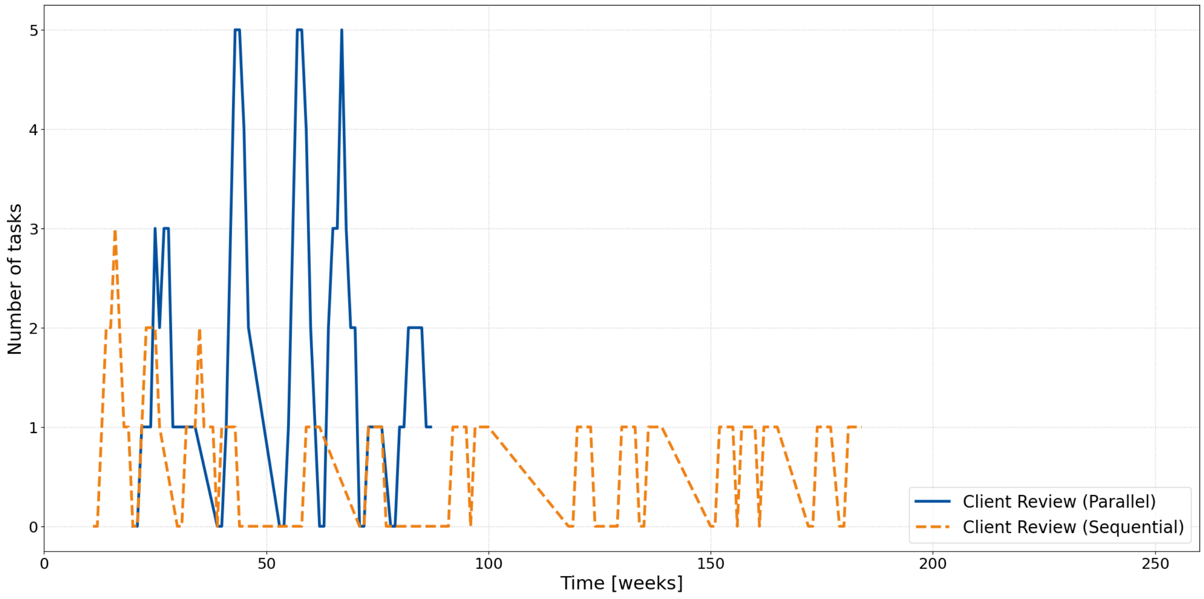

(a) Client review for parallel (blue) and sequential (orange) configurations.

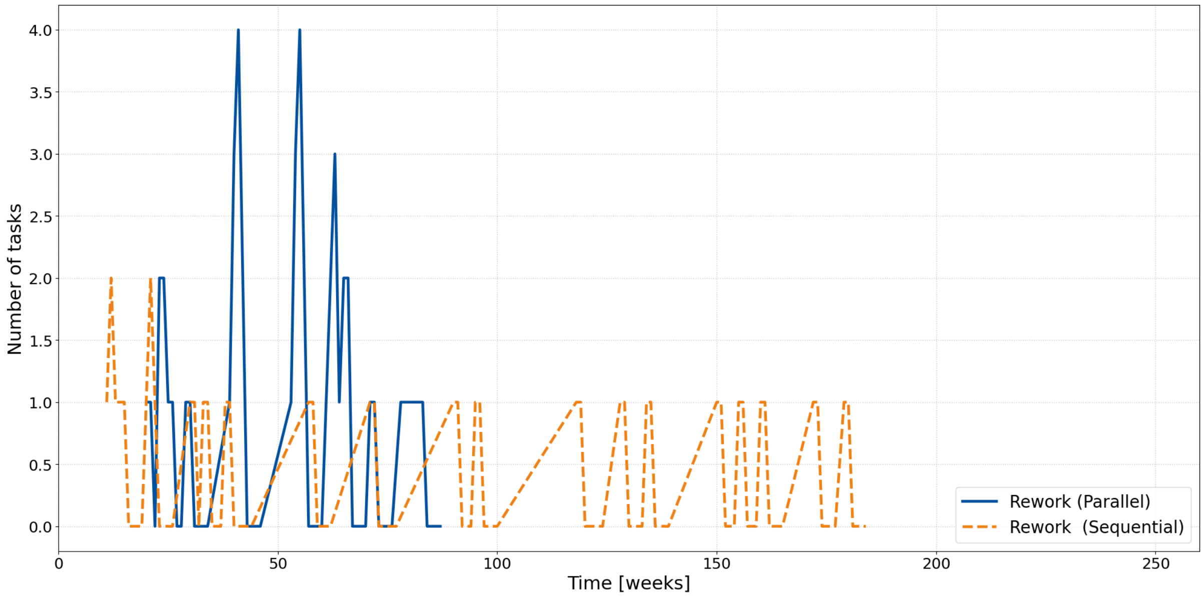

(b) Rework for parallel (blue) and sequential (orange) configurations.

Figure S2: Performance metrics for Experiment 1, two configurations of dependency: parallel and sequential. **(a)**, displays client review and **(b)**, represent rework.

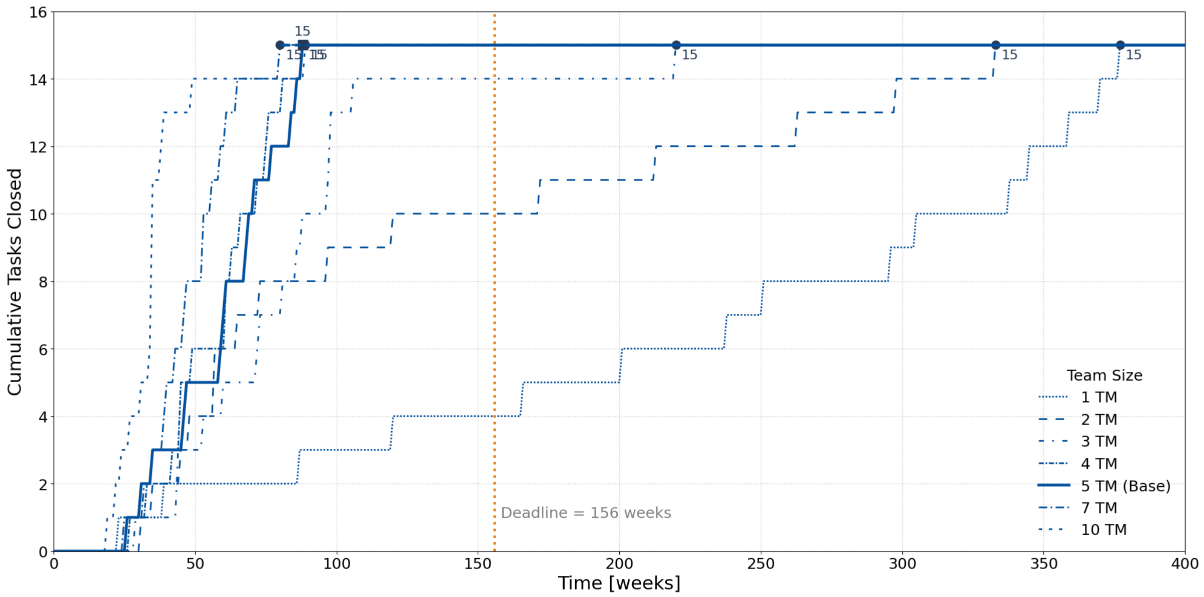

(a) Parallel

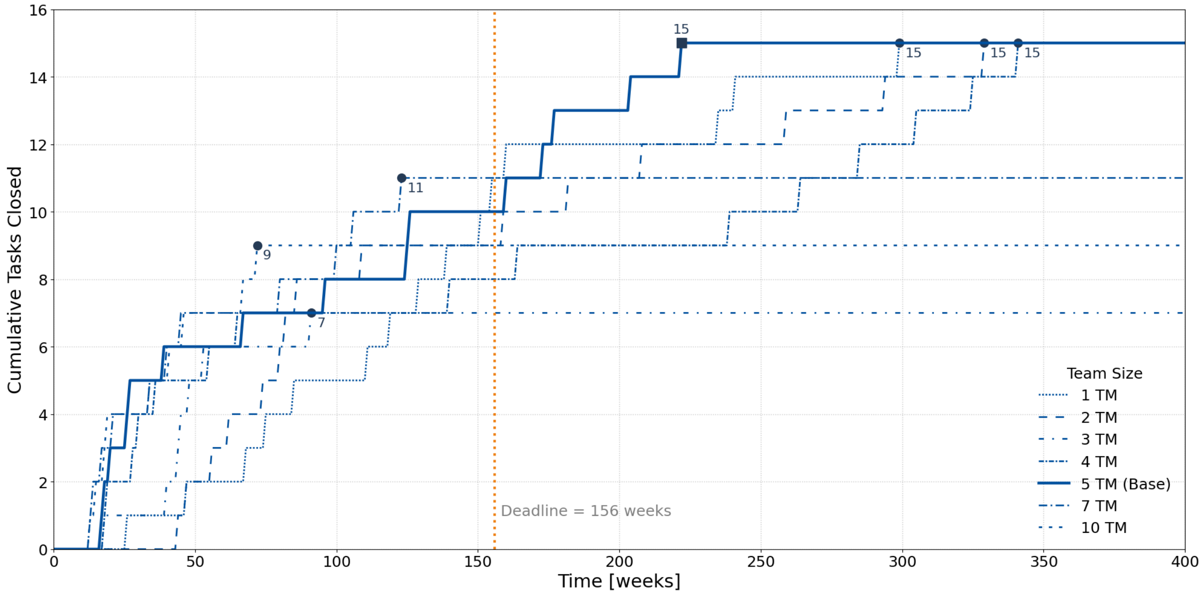

(b) Sequential

Figure S3: Cumulative number of closed tasks over time for different team sizes under two execution structures. **(a)** shows the parallel configuration, in which all tasks are independent and may be executed concurrently, whereas **(b)** represents the sequential configuration, where tasks are constrained by strict dependencies and executed in a fixed order. Each curve represents a simulation run for a specific team size: one, two, three, four, five (base case), seven, and ten team members. The vertical orange line indicates the project deadline, which is 156 weeks. Circle markers denote the completion time at which all 15 tasks are closed for each configuration. These plots illustrate how team capacity interacts with task structure to influence the timing and pacing of work completion.

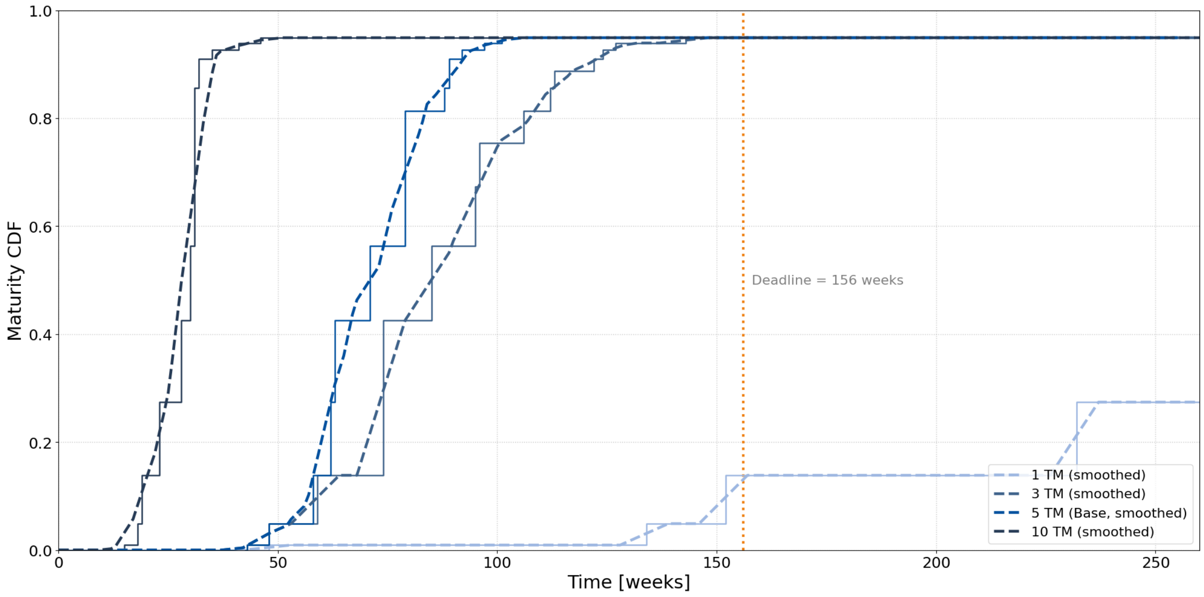

(a) Parallel

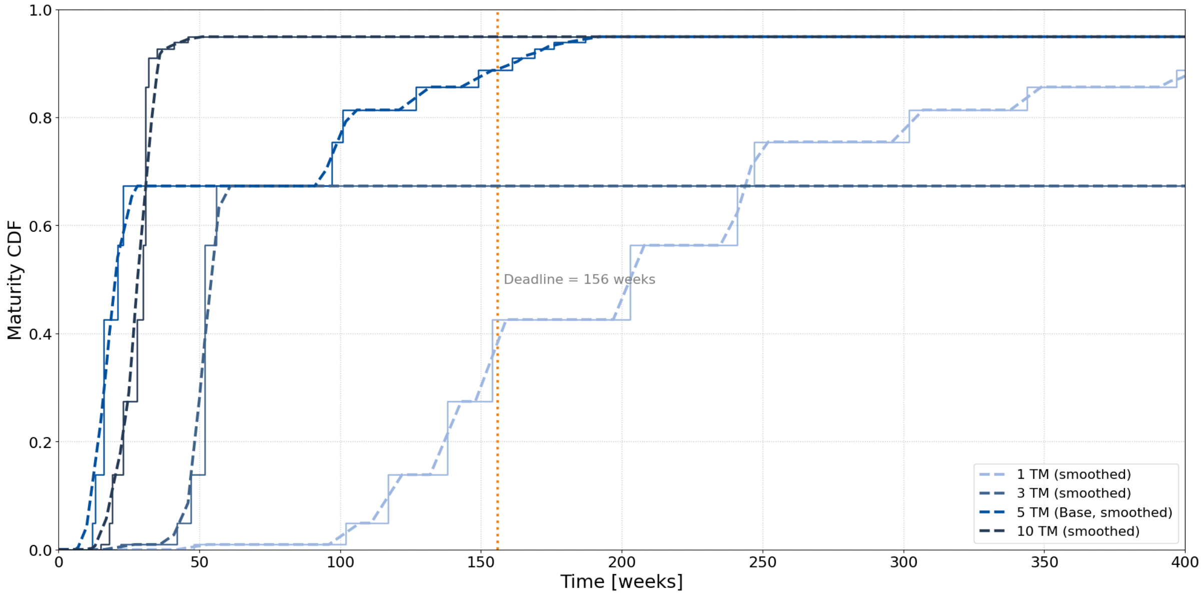

(b) Sequential

Figure S4: Cumulative maturity progression over time for selected team sizes in **(a)** parallel and **(b)** sequentilal task configurations. Curves represent smoothed maturity trajectories (CDF) for one, three, five, and ten team members. In the parallel case **(a)**, maturity increases rapidly with larger team sizes, converging toward full completion before the deadline. In contrast, the sequential case **(c)** exhibits delayed and plateaued maturity trajectories, particularly for team sizes of three and ten.

Table S6: Comparison between the NASA TRL framework [3] and the adapted TRL framework for chemical process industries, fragment from the study of [4, 5].

**TRL**	**Definitions, NASA**	**Definitions, Buchner et. al [4, 5]**
1	Basic principles observed and reported	Potential applications identified; conceptual design initiated based on scientific principles.
2	Technology concept and/or application formulated	Concept elaborated; preliminary performance demonstrated via analytical and experimental models. R&D activities planned.
3	Analytical and experimental critical function and/or characteristic proof-of-concept	Functional laboratory tests of prototype; qualitative validation of expected reaction or process behavior.
4	Component and breadboard validation in laboratory environment	Laboratory validation of functionality under controlled conditions; preliminary process development.
5	Component and breadboard validation in relevant environment	Controlled laboratory validation of components; scale-up to pilot level begins.
6	System/subsystem model or prototype demonstration in a relevant environment	Low-rate pilot plant operated; detailed process model derived; feasibility of integration assessed.
7	System prototype demonstration in the planned operational environment	Pilot plant performance optimized; equipment specifications validated for full-scale transfer.
8	Actual system completed and qualified through test and demonstration in the operational environment	Products/processes integrated into organizational structure; full-scale plant constructed.
9	Actual system proven through successful system and/or mission operations	Industrial-scale audited operations; enforceable performance guarantees confirmed.

References

[1] Transentis GmbH, Business prototyping toolkit for python (bptk-py), 2025. URL: https://github.com/transentis/bptk_py, accessed: 2025-05-27.

[2] Transentis Consulting, Business prototyping toolkit (bptk), 2025. URL: https://www.transentis.com/resources/business-prototyping-toolkit, accessed: 2025-05-27.

[3] J. C. Mankins, Technology readiness assessments: A retrospective, Acta Astronautica 65 (2009) 1216–1223.

[4] G. A. Buchner, A. W. Zimmermann, A. E. Hohgräve, R. Schomäcker, Techno-economic assessment framework for the chemical industry - based on technology readiness levels, Industrial and Engineering Chemistry Research 57 (2018) 8502–8517.

[5] G. A. Buchner, K. J. Stepputat, A. W. Zimmermann, R. Schomäcker, Specifying technology readiness levels for the chemical industry, 2019. doi:10.1021/acs.iecr.8b05693.
